# Structural Identification between Phthalazine-1,4-Diones and *N*-Aminophthalimides via Vilsmeier Reaction: Nitrogen Cyclization and Tautomerization Study

**DOI:** 10.3390/molecules26102907

**Published:** 2021-05-13

**Authors:** Cheng-Yen Chung, Ching-Chun Tseng, Sin-Min Li, Shuo-En Tsai, Hui-Yi Lin, Fung Fuh Wong

**Affiliations:** 1 School of Pharmacy, China Medical University, No. 91, Hsueh-Shih Rd., Taichung 40402, Taiwan; u109308001@cmu.edu.tw (C.-Y.C.); u106308201@cmu.edu.tw (C.-C.T.); u100003044@cmu.edu.tw (S.-E.T.); 2 The Ph.D. Program for Biotech Pharmaceutical Industry and School of Pharmacy, China Medical University, No. 91, Hsueh-Shih Rd., Taichung 40402, Taiwan; 3 Institute of New Drug Development, China Medical University, No. 91 Hsueh-Shih Rd., Taichung 40402, Taiwan; u102003483@cmu.edu.tw

**Keywords:** vilsmeier reagent, phthalazine-1,4-diones, *N*-aminophthalimide, hydrazine

## Abstract

*N*-Aminophthalimides and phthalazine 1,4-diones were synthesized from isobenzofuran-1,3-dione, isoindoline-1,3-dione, furo [3,4-*b*] pyrazine-5,7-dione, or 1*H*-pyrrolo [3,4-*c*] pyridine-1,3-dione with monohydrate hydrazine to carry out the 5-exo or 6-endo nitrogen cyclization under the different reaction conditions. Based on the control experimental results, 6-endo thermodynamic hydrohydrazination and kinetical 5-exo cyclization reactions were individually selective formation. Subsequently, Vilsmeier amidination derivatization was successfully developed to probe the structural divergence between *N*-aminophthalimide **2** and phthalazine 1,4-dione **3**. On the other hand, the best tautomerization of *N*-aminophthalimide to diazinone was also determined under acetic acid mediated solution.

## 1. Introduction

Nitrogen-containing heterocyclic compounds are widely applied to the biologically active pharmaceuticals, agrochemicals, and functional materials and become more and more important [1,2,3,4,5]. Especially, heterocycle derivatives containing bridgehead amine and hydrazine [6,7] such as *N*-aminophthalimides [8] and phthalazine 1,4-diones [9] have received considerable attention. Therefore, the development of new efficient methods to synthesize *N*-heterocycles with structural diversity is one major interest of modern synthetic organic chemists [10,11,12].

Heterocycles containing phthalazine 1,4-dione moiety have been reported to possess different pharmacological properties including anti-inflammatory, cardiotonic vasorelaxant, anticonvulsant [13], antihypertensive [14], antibacterial [15], anti-cancer [16], and carbonic anhydrase enzyme activity [17]. On the other hand, phthalimide group was conceived as a nitrogen source [18], for the direct introduction of masked amino function via the classical Gabriel protocol [19,20] as well as for the protection of amino groups [21,22,23]. *N*-aminophthalimides can be considered as phthalazine 1,4-dione tautomeric pairs. The structural arrangement of hydrazine derivatives is the mainly associated with the interconversion of imine−enamine [24,25]. Herein, we selectively synthesize *N*-aminophthalimide and phthalazine 1,4-dione derivatives in via the thermodynamic-kinetic control conditions. They will provide as the precursors for constructing the pharmacological heterocyclic compounds (PDE5 inhibitors) [26,27] or the chemiluminescent luminol derivatives [28]. Owing to the structural divergence between *N*-aminophthalimides **2** and phthalazine-1,4-diones **3**, we explored the Vilsmeier amidination derivatization to identify them in this work [29,30,31,32,33]. Furthermore, we successfully developed the prototrophic tautomeric interconversion from *N*-aminophthalimides to phthalazine 1,4-diones under Brønsted–Lowry acidic condition [29,30,31,32,33,34,35].

## 2. Results and Discussion

Initially, isobenzofuran-1,3-diones (**1a**–**c**), isoindoline-1,3-dione (**1d**), furo [3,4-*b*] pyrazine-5,7-dione (**1e**), and 1*H*-pyrrolo[3,4-*c*] pyridine-1,3-dione (**1f**) [34,35,36] were purchased or prepared as the starting materials. Reacting compounds **1a**–**f** with monohydrate hydrazine in ethanol solution at low (at 0 °C or –20 °C) or room temperature led directly to the 5-exo cyclization *N*-aminophthalimide products **2a**–**f** (81–94%) without the accumulation of hydrohydrazination products **3a**–**f** (Table 1). On the other hand, the 6-exo cyclization of distal nitrogen instead of proximal one was exclusively observed at reflux for ~4 h, and the corresponding 6-endo phthalazine 1,4-dione products **3a**–**f** were formed (83–91%, Table 1). Fortunately, compounds **2a**–**f** and **3a**–**f** can be selectively prepared via kinetic and thermodynamic control reaction with hydrazine hydrate. From the fundamental perspective, these symmetric molecules of *N*-aminophthalimide products **2a**–**f** provide themselves for investigation of tautomeric conversion controlling processes as well as convenient platforms for the structural identity.

**Figure 1:**
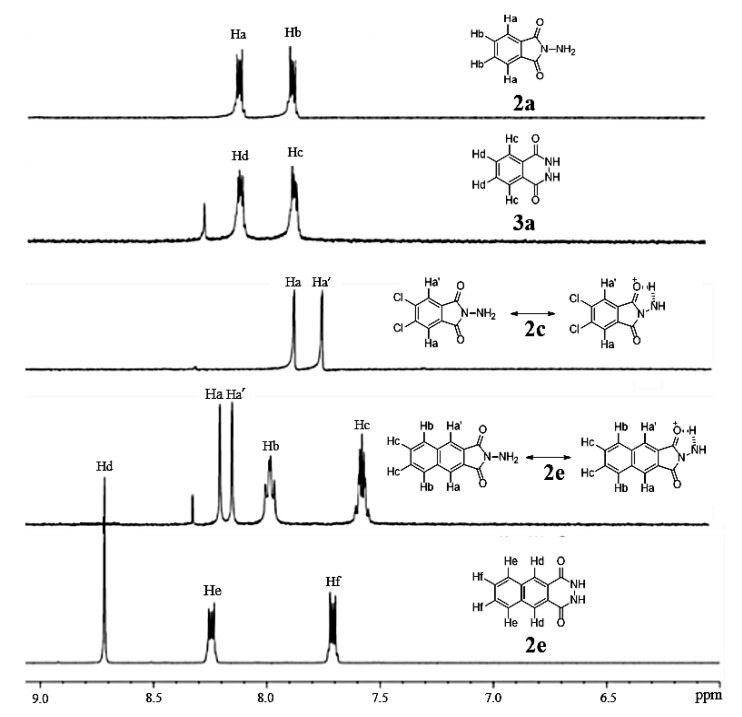
^1^H NMR spectra of *N*-aminophthalimides **2a**, **2c** and **2e** and phthalazines 1,4-dione **3a** and **3e**.

Additionally, phthalazine 1,4-diones **3a**–**f** were used as the reference standards. Although compounds **2** and **3** were tautomeric pairs, they significantly possessed the different polarity, such as, R_f_ = 0.33 for *N*-aminophthalimide **2a** and R_f_ = 0.41 for phthalazine 1,4-dione **3a** (EA/MeOH = 9/1). All of the **2a**–**f** and **3a**–**f** compounds were fully characterized by spectroscopic methods. For example, compound **2a** presented peaks at 3447 and 3482 cm^−1^ for stretching of the –NH_2_ group and at 1020 cm^−1^ for stretching of the N–N group in FT-IR spectrum. For compound **3a**, its IR absorption peaks were at 3461 cm^−1^ for stretching of the –NH–NH– group and at 1051 cm^−1^ for stretching of the N–N group. In ^1^H-NMR, compounds **2** and **3** presented similar chemical shift and coupling constants, resulting in difficult structural identification of each other. For example, the ^1^H-NMR spectra of compounds **2a** and **3a** were similar as shown in Figure 1. This observation drove us to develop a novel identification method.

**Figure 2:**
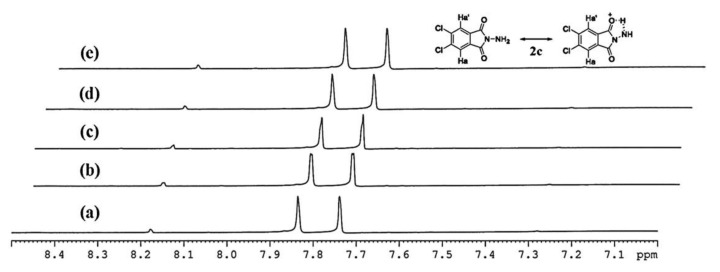
The timed programming result of *N*-aminophthalimides **2c** of ^1^H NMR spectra (**a**) 0 min, (**b**) 10 min, (**c**) 20 min, (**d**) 30 min, (**e**) 60 min.

On the other hand, *N*-aminophthalimides **2c** and **2e** reveal the existence of intramolecular hydrogen bonding phenomenon between carbonyl and amino group in ^1^H-NMR spectra. This phenomenon leads to the different chemical shift values between H_a_ and H_a’_ in an aromatic ring (Figure 1). However, compounds **3a**–**f** were favorable for the free base form in DMSO-*d*_6_ solvent. For example, the ^1^H-NMR spectrum of compound **3e** was presented in Figure 1. Furthermore, Compound **2c** was dissolved in DMSO-d6 and heated at ~100 °C by NMR technique. The sample was monitored in 0, 10, 20, 30, and 60 min, the timed programming result was shown in Figure 2, we found that the intramolecular hydrogen bonding phenomenon was very clearly stable.

**Scheme 1:**
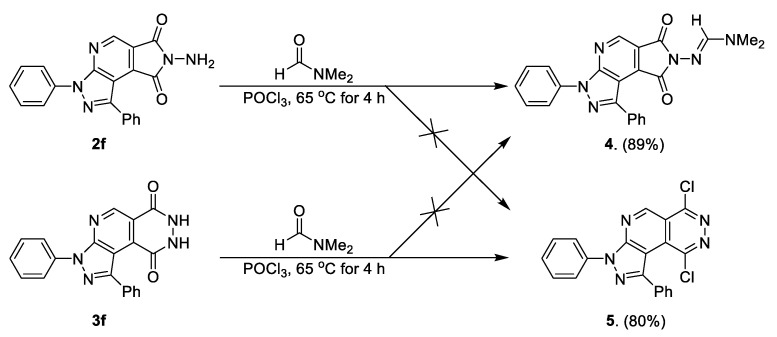
The results of 7-aminopyrazolopyrrolopyridine-6,8-dione **2f** and pyrazolopyridopyridazine dione **3f** treated with Vilsmeier reagent.

**Figure 3:**
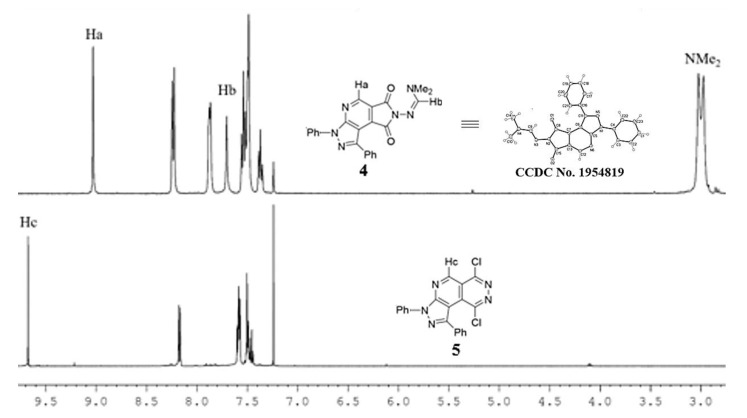
^1^H NMR spectra of 7-amidination product **4** and dichloropyridazine **5.**

Vilsmeier amidination methodology was essentially examined for the applicable protected utilization of primary amines. The usual method was directly treating primary amines with dimethylformamide (DMF) and coupling agents including POCl_3_, P_2_O_5_, PCl_5_, (COCl)_2_, PyBOP, SOCl_2_, acyl chlorides, trifluoroacetic anhydride (TFAA), or sulfonyl chloride to give the corresponding amidine products [37,38,39]. To further probe the structural divergence, pyrazolopyridopyridazine diones **2f** and *N*-aminopyrazolopyrrolopyridine-6,8-diones **3f** were selected as model cases for the further control experiments [28]. At first, we employed Vilsmeier reagent (halomethyleniminium salt) [29,30,31,32,33] to compounds **2f** and **3f** (Scheme 1). The reactions were individually monitored by TLC method. When compound **2f** was completely consumed for 4 h at 65 °C, the corresponding acquired amidination product **4** was formed and obtained in 89% yield without producing chlorinated compound **5**. The structure of compound **4** was fully characterized by spectroscopic methods and single-crystal X-ray diffraction study. Based on ^1^H NMR spectroscopic characterization, compound **4** possesses singlet signal of pyridine ring proton H_a_ around 9.03 ppm, and significant amidinyl moiety signals of iminium proton H_b_ around 7.70 ppm and two peaks of NMe_2_ around 2.97 and 3.02 ppm (Figure 3). These results showed the free primary amine group of compound **2f** was successfully converted into the amidinyl substituent. On the other hand, chlorination of compound **3f** was accomplished without amidination product **4** formation by Vilsmeier reagent at reflux for 4 h, affording the corresponding product **5** with down-field proton signal H_c_ of pyridine ring around 9.68 ppm in good yield (80%, Scheme 1 and Figure 3) [27]. Based on the above derivatization study, Vilsmeier reaction was conceived as the significant derivatization agent to identify isomers between **2f** and **3f**.

For further investigation into the reactivity of Vilsmeier amidination derivatization, Vilsmeier reaction was carried out using different substrates including *N*-aminophthalimides **2a**–**e** at 50 °C for 0.5 h. Various substituted reactants **2a**–**e** were demonstrated to perform the reactions smoothly, regardless of whether electron-donating or electron-withdrawing substituents, and the corresponding amidination products **6**−**10** were afforded in 74−88% yields (Table 2). All products **6**–**10** were fully characterized by spectroscopic methods, and they actually presented singlet peak for the significant amidinyl moiety signals of iminium proton H and two peaks of NMe_2_ in ^1^H-NMR. Subsequently, a series of phthalazine 1,4-diones **3a**–**e** were treated with Vilsmeier reagent (POCl_3_/DMF) at 65 °C or 80 °C for 2–4 h. The chlorination happened smoothly to afford the desired products **11**–**15** in high yields (82−90%, Table 2), except for **3d** (31%). Owing to the electron-rich property of nitrogen atoms on the aromatic motif of compound **3d**, the complicated aromatic substitution and polylization were proceeded. All chlorinated products **11**–**15** were also fully characterized by spectroscopic methods, and two peaks for the significant dione moieties were converted into –N = ^13^C–Cl singlet signal at δ 153–157 ppm in ^13^C-NMR spectrum. Therefore, Vilsmeier reagent (POCl_3_/DMF) was used as the derivatization reagent for the different reactive phenomenon to distinguish *N*-aminophthalimides **2** and phthalazine-1,4-diones **3**.

To explore the interconversion reactivity of the tautomerization, the solvent scope was first examined by using 7-aminopyrazolopyrrolopyridine-6,8-dione **2f**. Compound **2f** was screened and refluxed in the various solvents including CH_2_Cl_2_, THF, EtOH, MeCN, toluene, dioxane, and DMSO for 24 h. However, the reactions in CH_2_Cl_2_, EtOH, toluene recovered the starting material **2f** without conversion happening (Table 3 entries 1–3). The use of polar THF, MeCN, dioxane, and DMSO led to lower interconversion ratios of **2f**/**3f** from 93/7 to 88/12 (Table 3, entries 4–7). Subsequently, Brønsted–Lowry acids including acetic acid (AcOH), methanesulfonic acid (TsOH), methanesulfonic chloride (TsCl), and trifluoroacetic acid (TFA) were studied for the interconversion reaction at reflux for 4 h (Entries 8–11, Table 3). Several experimental observations are worthy to discuss: we firstly found that the conversion ratios were improved under acidic condition (Entries 8–11, Table 3). Secondly, under the strong acid such as trifluoroacetic acid (pKa = 0.30), *p*-toluenesulfonic acid (TsOH, pKa = −1.9), and methanesulfonic chloride (TsCl), the low conversion ratio and decomposed products were observed (Entries 8–10, Table 3).

**Figure 4:**
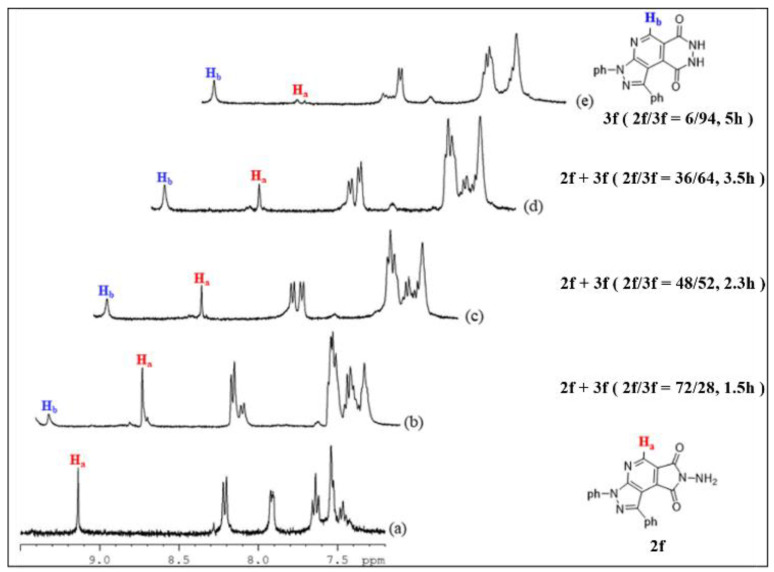
(**a**) ^1^H NMR spectrum of the beginning of the reaction (^1^H NMR of compound **2f**). (**b**–**d**) Reaction at reflux for 1.5, 2.3, and 3.5 h (^1^H NMR of compounds **2f** and **3f**; the ratios of **2f**/**3f** = ~72/28, 48/52, and 36/64). (**e**) Reaction at reflux for 5 h (^1^H NMR of compounds **3f**; the ratios of **2f**/**3f** = ~6/94).

For further investigations, the timed programming of the thermodynamic conversion of compound **2f** was carried out under acetic acid (AcOH) solution and shown in Figure 4. The reaction mixture was sampled at 1.5, 2.3, 3.5, and 5 h and detected by the ^1^H-NMR spectroscopic method. This result showed that compound **2f** was gradually converted to the thermodynamic stable product **3f** (Figure 4). Finally, transformation reaction was equilibrated at reflux for more than 5 h, and the conversion ratio was obtained proximately 6/94 (**2f**/**3f**, Entry 11 of Table 4 and Figure 4). Based on the above experimental result, acetic acid was conceived as the best acidic solvent with 6/94 conversion ratio. Fortunately, **2f** can be successfully and smoothly transformed to more thermodynamically stable product **3f** by refluxing in acidic medium [40,41]. To further demonstrate the reliable of conversion procedure, *N*-aminophthalimides **2a**–**e** were also used as starting materials at reflux for 8–9 h. Fortunately, compounds **2a**–**e** can be smoothly transformed to give the corresponding thermodynamically pyrazolopyridopyridazine diones **3a**–**e** under acetic acid solvent, with the ratio of **2a**–**e**/**3a**–**e** from 6/94 to 1/99 (Table 4).

## 3. Experimental Section

### 3.1. General Procedure

All reagents were purchased commercially. All reactions were carried out under argon or nitrogen atmosphere and monitored by TLC. Flash column chromatography was carried out on silica gel (230–400 mesh). Analytical thin-layer chromatography (TLC) was performed using pre-coated plates (silica gel 60 F-254) purchased from Merck Inc. Flash column chromatography purification was carried out by gradient elution using *n*-hexane in ethyl acetate (EtOAc) unless otherwise stated. ^1^H NMR spectra were recorded at 400 or 500 MHz and ^13^C NMR spectra were recorded at 100 or 125 MHz, respectively, in CDCl_3_, DMSO-*d*_6_, or D_2_O solvent. The standard abbreviations s, d, t, q, and m refer to the singlet, doublet, triplet, quartet, and multiplet, respectively. Coupling constant (*J*), whenever discernible, has been reported in Hz. Infrared spectra (IR) were recorded in neat solutions or solids; and mass spectra were recorded using electron impact or electrospray ionization techniques. The reported wavenumbers are referenced to the polystyrene 1601 cm^−1^ absorption. ESI-MS analyses were performed on an Applied Biosystems API 300 mass spectrometer. High-resolution mass spectra were obtained from a JEOL JMS-HX110 mass spectrometer.

### 3.2. Standard Procedure for Synthesis of N-Aminophthalimides ***2a**–**f***

Standard procedure for synthesis of *N*-aminophthalimides **2a**–**f.** The reliable procedure involved the treatment of isobenzofuran-1,3-diones (**1a**–**c**), isoindoline-1,3-dione (**1d**), furo[3,4-*b*] pyrazine-5,7-dione (**1e**), or 1*H*-pyrrolo[3,4-*c*] pyridine-1,3-dione (**1f**, 1.0 equiv.) with monohydrate hydrazine (~5.0 equiv.) in EtOH/H_2_O (2.0 mL/2.0 mL) at 0 °C or –20 °C to room temperature within 4 h. When the reaction was completed, the reaction mixture was added water (10 mL) for precipitation. The precipitate was filtered, washed with cold water (10 mL) and *n*-hexane/EA (1/2, 15 mL) to give the corresponding crude *N*-aminophthalimides **2a–f**. The crude desired products **2a–f** were recrystallized in acetone/THF (1/4) solution to obtain the pure *N*-aminophthalimides **2a–f** in 81–94% yields. The low solubility of the compounds **2a–f** made the ^13^C-NMR characterization of quaternary and carbonyl carbons of these substrates unclear [28].

*N-Aminophthalimide* (**2a**) [42]: White solid; 92% yield; mp 202–205 °C; ^1^H NMR (DMSO-*d*_6_, 400 MHz) δ 7.83 (dd, *J* = 5.8, 3.4 Hz, 2H, Ar*H*), 8.05 (dd, *J* = 5.8, 3.4 Hz, 2H, Ar*H*); ^13^C NMR (DMSO-*d*_6_, 100 MHz): δ 123.28 (2 × C), 130.53 (2 × C), 134.81 (2 × C), 167.36 (2 × C); FT-IR (KBr): 3482, 3341, 3262, 3093, 1778, 1719, 1605, 1468, 1409, 1291, 1197, 1091, 1075, 1020, 918, 800, 718, 526 cm^−1^; EIMS *m/z*: 162 (M^+^, 100), 105 (12), 104 (74), 77 (12), 76 (26), 50 (11); HRMS (EI) *m/z*: [M]^+^ Calcd for C_8_H_6_N_2_O_2_: 162.0429; Found: 162.0425.

*N-Amino-4,5-difluoropthalimide* (**2b**): white solid; 92% yield; mp 288–290 °C; ^1^H NMR (D_2_O, 500 MHz): δ 7.40 (t, *J* = 9.38 Hz, 2H, Ar*H*); ^13^C NMR (D_2_O, 125 MHz): δ 116.43 (dd, *J* = 12.98, 6.09 Hz, 2 × C), 134.91 (2 × C), 148.62 (d, *J* = 14.79 Hz), 150.61 (d, *J* = 14.78 Hz), 175.06 (2 × C). FT-IR (KBr): 3358, 3290, 3072, 2593, 1658, 1597, 1495, 1372, 1182, 1094, 1029, 910, 788, 743 cm^−1^; EIMS *m/z*: 198 (M^+^, 100), 141 (24), 140 (83), 113 (14), 112 (33); HRMS (EI) *m/z*: [M]^+^ Calcd for C_8_H_4_F_2_N_2_O_2_: 198.0241; Found: 198.0233.

*N-Amino-4,5-dichlorophthalimide* (**2c**): White solid; 81% yield; mp 302–305 °C; ^1^H NMR (DMSO-*d*_6_, 400 MHz) δ 7.72 (s, 1H, Ar*H*), 7.84 (s, 1H, Ar*H*); ^13^C NMR (DMSO-*d*_6_, 100 MHz) δ 130.36, 131.12, 132.08, 132.25, 132.57, 141.90, 164.74, 169.22; FT-IR (KBr): 3466, 3321, 3220, 3027, 2646, 1653, 1619, 1521, 1393, 1356, 1308, 1106, 1022, 930, 809, 782, 613 cm^−1^; EIMS *m/z*: 232 (M^+^ + 2, 64), 230 (M^+^, 100), 174 (46), 173 (12), 172 (72), 146 (13), 144 (19), 109 (19), 74 (17); HRMS *m/z*: [M]^+^calcd for C_8_H_4_Cl_2_N_2_O_2_: 229.9650; found: 229.9653.

*N-Amino-2,3-pyrazinedicarboxylicphthalimide* (**2d**) [42]: Brown solid; 82% yield; mp 220–222 °C; ^1^H NMR (D_2_O, 400 MHz) δ 8.59 (s, 2H, Ar*H*); ^13^C NMR (D_2_O, 100 MHz) δ 143.28 (2 × C), 149.18 (2 × C), 172.29 (2 × C); FT-IR (KBr): 3430, 3282, 3164, 2936, 2750, 2636, 1679, 1621, 1353, 1161, 1107, 975, 825 cm^−1^. EIMS *m/z*: 164 (M^+^, 36), 150 (25), 124 (40), 106 (76), 80 (100), 79 (30), 78 (56), 53 (69), 52 (95), 51 (52). HRMS *m/z*: [M]^+^calcd for C_6_H_4_N_4_O_2_: 164.0334; found: 164.0338.

*Naphthalene-2,3-dicarboxylic hydrazide* (**2e**): Yellow solid; 94% yield; mp 298–300 °C; ^1^H NMR (DMSO-*d*_6_, 400 MHz) δ 7.51–7.56 (m, 2H, Ar*H*), 7.92–7.96 (m, 2H, Ar*H*), 8.11 (s, 1H, Ar*H*), 8.16 (s, 1H, Ar*H*); ^13^C NMR (DMSO-*d*_6_, 100 MHz) δ 127.12, 127.52, 128.32, 128.48, 128.68, 128.87, 132.17, 132.32, 133.31, 137.68, 168.21, 171.85; FT-IR (KBr): 3466, 3429, 3304, 3190, 3054, 2653, 1673, 1636, 1606, 1390, 1339, 1177, 1140, 1099, 964, 809, 758, 481 cm^−1^; EIMS *m/z*: 213 (14), 212 (M^+^, 100), 155 (10), 154 (45), 127 (14), 126 (39); HRMS *m/z*: [M]^+^calcd for C_12_H_8_N_2_O_2_: 212.0586; found: 212.0578.

*7-Amino-1,3-diphenylpyrazolo[3,4-b]pyrrolo[3,4-d]pyridine-6,8(3H,7H)-dione* (**2f**) [28]: White solid; 83% yield; mp 217–219 °C; ^1^H NMR (DMSO-*d*_6_, 400 MHz) δ 4.55 (br, 2H, N*H*_2_), 7.42 (t, *J* = 7.46 Hz, 1H, Ar*H*), 7.49–7.55 (m, 3H, Ar*H*), 7.60–7.65 (m, 4H, Ar*H*), 8.25 (d, *J* = 8.08 Hz, 2H, Ar*H*), 8.82 (s, 1H, Ar*H*).

### 3.3. Standard Procedure for Synthesis of Phthalazine 1,4-Diones ***3a**–**f***

The reliable procedure involved the treatment of isobenzofuran-1,3-diones (**1a**–**c**), isoindoline-1,3-dione (**1d**), furo[3,4-*b*] pyrazine-5,7-dione (**1e**), or 1*H*-pyrrolo[3,4-*c*] pyridine-1,3-dione (**1f**, 1.0 equiv.) with monohydrate hydrazine (~40 equiv.) in EtOH solution (2.0 mL) at room temperature or in neat at reflux for 4 h. When the reaction was completed, the reaction mixture was added water (10 mL) for precipitation. The precipitation was filtered, washed with cold water (10 mL) and *n*-hexane/EA (1/2, 15 mL) to give the corresponding crude phthalazine 1,4-diones **3a–f**. The crude desired products **3a–f** were recrystallized in acetone/THF (1/4) solution to obtain the pure phthalazine 1,4-diones **3a–f** in 83–91% yields. The low solubility of the compounds **3a–f** made the ^13^C-NMR characterization of quaternary and carbonyl carbons of these substrates unclear [28].

*2,3-Dihydro-phthalazine-1,4-dione* (**3a**) [43]: White solid; 89% yield; mp 227–229 °C; ^1^H NMR (DMSO-*d*_6_, 400 MHz) δ 7.82 (dd, *J* = 5.93, 3.30 Hz, 2H, Ar*H*), 8.06 (dd, *J* = 5.89, 3.28 Hz, 2H, Ar*H*); ^13^C NMR (DMSO-*d*_6_, 100 MHz) δ 125.78 (2 × C), 128.68 (2 × C), 132.35 (2 × C), 156.41 (2 × C); FT-IR (KBr): 3461, 3207, 2715, 1773, 1749, 1609, 1468, 1386, 1309, 1051, 715 cm^−1^.

*6,7-Fluoroo-2,3-dihydrophthalazine-1,4-dione* (**3b**): White solid; 85% yield; mp 220–222 °C; ^1^H NMR (DMSO-*d*_6_, 400 MHz) δ 7.99 (t, *J* = 9.11 Hz, 2H, Ar*H*); ^13^C NMR (DMSO-*d*_6_, 100 MHz) δ 114.38 (2 × C), 126.58 (2 × C), 151.48 (d, *J* = 16.11 Hz), 154.02 (d, *J* = 16.10 Hz), 154.61 (2 × C); FT-IR (KBr): 3490, 3180, 3070, 2610, 1660, 1590, 1511, 1460, 1354, 1303, 1189, 1067, 899, 804, 565 cm^−1^; EIMS *m/z*: 199 (13), 198 (M^+^, 100), 141 (23), 140 (75), 113 (17), 112 (30), 63 (13); HRMS *m/z*: [M]^+^calcd for C_8_H_4_F_2_N_2_O_2_: 198.0241; found: 198.0234.

*6,7-Dichloro-2,3-dihydrophthalazine-1,4-dione* (**3c**): White solid; 87% yield; mp 237–239 °C; ^1^H NMR (DMSO-*d*_6_, 400 MHz) δ 8.15 (s, 2H, Ar*H*); ^13^C NMR (DMSO-*d_6_*, 100 MHz) δ 128.16 (2 × C), 129.64 (2 × C), 134.98 (2 × C), 156.12 (2 × C); FT-IR (KBr): 3314, 3193, 2984, 2926, 2879, 1666, 1565, 1467, 1450, 1373, 1075, 822 cm^−1^; EIMS *m/z*: 232 (M^+^ + 2, 64), 231 (M^+^ + 1, 17) 230 (M^+^, 100), 174 (46), 173 (12), 172 (72), 146 (13), 144 (19), 109 (19), 74 (17); HRMS *m/z*: [M]^+^calcd for C_8_H_4_Cl_2_N_2_O_2_: 229.9650; found:229.9643.

*7-Dihydropyrazino[2,3-d] pyridazine-5,8-dione* (**3d**) [44]: Brown solid; 83% yield; mp 325–327 °C; ^1^H NMR (D_2_O, 500 MHz) δ 8.82 (s, 2H, ArH); ^13^C NMR (D_2_O, 125 MHz) δ 145.14 (2 × C), 146.29 (2 × C), 169.64 (2 × C). FT-IR (KBr): 3343, 3204, 1661, 1632, 1543, 1314, 1293, 1100 cm^−1^; EIMS *m/z*: 164 (M^+^, 1), 150 (26) 140 (16), 106 (77), 80 (100), 78 (46), 52 (2).

*2,3-Dihydrobenzo[g]phthalazine-1,4-dione* (**3e**): White solid; 91% yield; mp 301–303 °C; ^1^H NMR (DMSO-d6, 400 MHz) δ 7.71(*dd*, *J* = 6.29, 3.27 Hz, 2H, ArH), 8.25 (*dd*, *J* = 6.28, 3.28 Hz, 2H, ArH), 8.72 (s, 2H, ArH); ^13^C NMR (DMSO-*d6*, 100 MHz) δ 125.13 (2 × C), 126.69 (2 × C), 128.84 (2 × C), 129.66 (2 × C), 134.54 (2 × C), 156.22 (2 × C); FT-IR (KBr): 3416, 1663, 1626, 1501, 1467, 1440, 1366, 1072, 815 cm^−1^; EIMS *m/z*: 213 (14), 212 (M+, 100), 155 (12), 154 (43), 237 (17), 126 (44); HRMS *m/z*: [M]^+^calcd for C_12_H_8_N_2_O_2_: 212.0586; found: 212.0588.

*1,3-Diphenyl-7,8-dihydro-3H-pyrazolo [4’,3’:5,6] pyrido[3,4-d] pyridazine-6,9-dione(**3f**)* [28]: white solid; 84% yield; mp 292–295 °C; ^1^H NMR (DMSO-*d*_6_, 600 MHz) δ 7.43–7.47 (m, 4H, Ar*H*), 7.60–7.64 (m, 4H, Ar*H*), 8.20 (d, *J* = 7.88 Hz, 2H, Ar*H*), 9.43 (s, 1H, Ar*H*).

### 3.4. Standard Procedure for Preparation of Amidination Products ***4*** and ***6**–**10*** from N-Aminophthalimides ***2a**–**f*** with Vilsmeier Reagent (POCl_3_/DMF)

The reliable procedure that involved *N*-aminophthalimides **2a**–**f** (1.0 equiv..) was individually treated with ~3.0 equivalent amount of POCl_3_ in *N*, *N*-dimethylformamide solution (DMF, 2.0 mL) at 50 °C or 65 °C for 0.5–4 h. When the reaction was completed, the reaction mixture was added to saturate sodium bicarbonate (15 mL) and extracted with dichloromethane (15 mL). The organic extracts were dried over MgSO_4_, filtered, and concentrated under reduced pressure. The residues were purified by column chromatography on silica gel to give the corresponding amidination products **4** in 89% yield and **6**–**10** in 74–88% yields [37,38,39].

*N’-(6,8-Dioxo-1,3-diphenyl-6,8-dihydropyrazolo[3,4-b]pyrrolo[3,4-d]pyridin-7(3H)-yl)-N,N-dimethylformimidamide* (**4**): Brown solid; 89% yield; mp 225–227 °C; ^1^H NMR (CDCl_3_, 400 MHz) δ 2.97 (s, 3H, NMe), 3.02 (s, 3H, NMe), 7.37 (t, *J* = 7.36 Hz, 1H, Ar*H*), 7.48–7.50 (m, 3H, Ar*H*), 7.54 (t, *J* = 7.74 Hz, 2H, Ar*H*), 7.70 (s, 1H, Ar*H*), 7.87 (d, *J* = 4.40 Hz, 2H, Ar*H*), 8.24 (d, *J* = 8.00 Hz, 2H, Ar*H*), 9.03 (s, 1H, Ar*H*); ^13^C NMR (CDCl_3_, 100 MHz) δ 33.83, 41.06, 108.85, 119.70, 122.21 (2 × C), 127.20, 127.90 (2 × C), 129.14 (2 × C), 129.37, 129.82 (2 × C), 131.52, 133.65, 138.40, 143.04, 146.08, 153.77, 161.77, 163.79, 165.45; FT-IR (KBr): 3064, 2923, 2852, 1714, 1626, 1500, 1413, 846 cm^−1^; EIMS *m/z*: 411 (25), 410 (M^+^, 100), 341 (29), 340 (87), 339 (52), 268 (14), 77 (31); HRMS calcd. For C_23_H_18_N_6_O_2_: 410.1491; found: 410.1482.

*N’-(1,3-Dioxo-1,3-dihydro-2H-isoindol-2-yl)-N,N-dimethyliminoformamide hydrochloride* (**6**): Yellow solid; 88% yield; mp 177–179 °C; ^1^H NMR (CDCl_3_, 400 MHz) δ 3.02 (s, 6H, N(CH_3_)_2_), 7.66 (dd, *J* = 5.37, 3.03, 2H, Ar*H*), 7.75 (s, 1H), 7.79 (dd, *J* = 5.42, 3.09, 2H, Ar*H*); ^13^C NMR (CDCl_3_, 100 MHz) δ 34.84, 41.10, 123.01 (2 × C), 123.58 (2 × C), 130.77 (2 × C), 133.77 (2 × C), 161.52, 166.38 (2 × C); FT-IR (KBr): 3446, 2933, 1699, 1620, 1321, 1138, 706 cm^−1^; EIMS *m/z*: 218 (12), 217 (M^+^, 100), 148 (19), 130 (27), 105 (17), 104 (22), 90 (11), 76 (29), 71 (41), 70 (21); HRMS *m/z*: [M]^+^calcd for C_11_H_11_N_3_O_2_: 217.0851; found: 217.0842.

*N’-(1,3-Dioxo-5,6-difluoro-2H-isoindolin-2-yl)-N,N-dimethylformimidamide* (**7**): Light yellow solid; 74% yield; mp 183–185 °C; ^1^H NMR (CDCl_3_, 400 MHz) δ 2.88 (s, 3H, NMe), 2.96 (s, 3H, NMe), 7.69 (t, *J* = 8.96 Hz, 2H, Ar*H*), 8.04 (s, 1H); ^13^C NMR (CDCl_3_, 100 MHz) δ 31.83, 36.98, 119.75 (dd, *J* = 13.4, 7.63 Hz, 2 × C), 129.46 (t, *J* = 4.77 Hz, 2 × C), 150.11 (d, *J* = 14.45 Hz), 152.68 (d, *J* =14.31 Hz), 163.68, 168.39 (2 × C); FT-IR (KBr): 3048, 2919, 1695, 1620, 1450, 1355, 1148, 818 cm^−1^; EIMS *m/z*: 254 (13), 253 (M^+^, 100), 166 (20), 141 (25), 140 (40), 139 (16), 126 (22), 125 (12), 113 (13), 112 (52), 111 (20), 109 (16), 97 (23), 95 (17), 85 (16), 83 (21), 81 (17); HRMS *m/z*: [M]^+^calcd for C_11_H_9_F_2_N_3_O_2_: 253.0663; found: 253.0657.

*N’-(1,3-Dioxo-5,6-dichloro-2H-isoindolin-2-yl)-N,N-dimethylformimidamide* (**8**): Light orange solid; 85% yield; mp 192–194 °C; ^1^H NMR (CDCl_3_, 400 MHz) δ 3.04 (s, 6H, N(C*H*_3_)_2_), 7.74 (s, 1H), 7.87 (s, 2H, Ar*H*); ^13^C NMR (CDCl_3_, 100 MHz) δ 34.80, 41.07, 125.09 (2 × C), 129.90 (2 × C), 138.54 (2 × C), 161.35, 164.42 (2 × C). FT-IR (KBr): 3092, 3024, 2926. 1773, 1705, 1624, 1345, 1145, 774 cm^−1^; EIMS *m/z*: 287 (M^+^ + 2, 61), 286 (M^+^ +1, 12), 285 (M^+^, 100), 198 (13), 175 (12), 174 (14), 173 (22), 172 (20), 146 (16), 144 (24), 109 (11), 71 (98), 70 (36), 69 (10); HRMS *m/z*: [M]^+^calcd for C_11_H_9_Cl_2_N_3_O_2_: 285.0072; found: 285.0064.

*N’-(1,3-Dioxo-5,7-dihydro-6H-pyrrolo[3,4-b]pyrazin-6-yl)-N,N-dimethylformimidamide* (**9**): Light yellow solid; 88% yield, mp 219–221 °C; ^1^H NMR (CDCl_3_, 400 MHz) δ 3.03 (s, 3H, NMe), 3.05 (s, 3H, NMe), 7.81 (s, 1H), 8.84 (s, 2H, Ar*H*); ^13^C NMR (CDCl_3_, 100 MHz) δ 34.83, 41.18, 146.17 (2 × C), 148.52 (2 × C), 161.28, 162.31 (2 × C); FT-IR (KBr): 1654, 1289, 1545, 1293, 1104, 825 cm^−1^; EIMS *m/z*: 220 (12), 219 (M^+^, 56), 193 (10), 179 (13), 178 (13), 169 (12), 168 (12), 167 (14), 165 (11), 155 (11), 152 (11), 151 (26), 150 (14), 149 (23), 147 (11), 141 (12), 139 (15), 137 (13), 135 (11), 127 (12), 125 (21), 123 (19), 121 (11), 119 (11), 115 (14), 113 (14), 112 (14), 111 (36), 110 (11), 109 (27), 107 (15), 106 (16), 105 (16), 99 (17), 98 (13), 97 (52), 96 (16), 95 (36), 93 (12), 91 (27); HRMS *m/z*: [M]^+^calcd for C_9_H_9_N_5_O_2_: 219.0756; found: 219.0748.

*N’-(1,3-Dioxo-5,6-dihydro-2H-benzo[f]isoindol-2-yl)-N,N-dimethylformimidamide* (**10**): Light yellow solid; 77% yield; mp 199–201 °C; ^1^H NMR (CDCl_3_, 400 MHz) δ 3.05 (s, 3H, NMe), 3.07 (s, 3H, NMe), 7.64–7.69 (m, 2H, Ar*H*), 7.84 (s, 1H), 7.99–8.02 (m, 2H, Ar*H*), 8.28 (s, 2H, Ar*H*); ^13^C NMR (CDCl_3_, 100 MHz) δ 34.89, 41.10, 124.33 (2 × C), 126.68 (2 × C), 128.99 (2 × C), 130.20 (2 × C), 135.45 (2 × C), 161.23, 166.00 (2 × C) cm^−1^; FT-IR (KBr): 2918, 2807, 1757, 1693, 1621, 1521, 1425, 1411, 1321, 1154, 1118, 1004, 900, 754, 479 cm^−1^; EIMS *m/z*: 268 (19), 267 (M^+^, 100), 225 (13), 210 (10), 198 (26), 197 (49), 180 (28), 155 (64), 154 (24), 153 (20), 152 (13), 140 (21), 127 (26), 126 (74), 71 (17), 57 (11); HRMS *m/z*: [M]^+^calcd for C_15_H_13_N_3_O_2_: 267.1008; found: 267.1015.

### 3.5. Standard Procedure for Preparation of Chlorination Products ***5***, and ***11**–**15*** from Phthalazine 1,4-Diones ***3a**–**f*** with Vilsmeier Reagent (POCl_3_/DMF)

The reliable procedure that involved phthalazine 1,4-diones **3a**–**f** (1.0 equiv.) was individually treated with ~3.0 equivalent amount of POCl_3_ in *N*, *N*-dimethylformamide solution (DMF, 2.0 mL) at 65 °C or 80 °C for 2–4 h. When the reaction was completed, the reaction mixture was added to saturate sodium bicarbonate (15 mL) and extracted with dichloromethane (15 mL). The organic extracts were dried over MgSO_4_, filtered, and concentrated under reduced pressure. The residues were purified by column chromatography on silica gel to give the corresponding chlorinated products **5** in 80% yield and **11**–**15** in 31–90% yields [29].

*6,9-Dichloro-1,3-diphenyl-3H-pyrazolo[4’,3’:5,6]pyrido[3,4-d]pyridazine* (**5**): Light yellow solid; 80% yield; mp 196–197 °C; ^1^H NMR (CDCl_3_, 500 MHz) δ 7.46 (t, *J* = 7.43 Hz, 1H, Ar*H*), 7.49–7.51 (m, 3H, Ar*H*), 7.57–7.60 (m, 4H, Ar*H*), 8.17 (dd, *J* = 8.60, 1.09 Hz, 2H, Ar*H*), 9.68 (s, 1H, Ar*H*); ^13^C NMR (CDCl_3_, 125 MHz) δ 103.92, 118.46, 123.39 (2 × C), 127.91, 127.95 (2 × C), 128.19, 129.13, 129.33 (2 × C), 130.01 (2 × C), 135.22, 137.87, 147.79, 149.88, 151.00, 152.05, 153.49; FT-IR (KBr): 3064, 2923, 2846, 1571, 1501, 1413, 1243, 1119, 863 cm^−1^; EIMS *m/z*: 395 (12), 394 (17), 393 (M^+^ + 2, 65), 392 (36), 391 (M^+^, 99), 390 (20), 356 (20), 321 (35), 320 (60), 288 (14), 263 (12), 244 (33), 218 (17), 91 (19), 77 (100); HRMS calcd. For C_20_H_11_Cl_2_N_5_: 391.0392; found: 391.0397.

*1,4-Dichlorophthalazine* (**11**): Yellow solid; 90% yield; mp 162–164 °C; ^1^H NMR (CDCl_3_, 400 MHz) δ 7.74–7.76 (m, *2*H, Ar*H*), 7.85–7.7.87 (m, *2*H, Ar*H*); ^13^C NMR (CDCl_3_, 100 MHz) δ 125.86 (2 × C), 127.21 (2 × C), 134.49 (2 × C), 155.03 (2 × C); IR (KBr): 3157, 3000, 2896, 2875, 1671, 1346, 1289, 1157, 993, 775, 664 cm^−1^; EIMS *m/z*: 202 (M^+^ + 4, 10), 200 (M^+^ + 2, 63), 198 (M^+^, 100), 182 (25), 180 (77), 172 (17), 170 (26), 151 (17), 135 (20), 128 (14), 125 (11), 123 (29), 102 (17), 101 (11), 99 (20), 90 (11). HRMS calcd. For C_8_H_4_Cl_2_N_2_: 197.9752; found: 197.9746.

*1,4-Dichloro-2,3-difluorophthalazine* (**12**): White solid; 82% yield; mp 75–76 °C; ^1^H NMR (CDCl_3_, 400 MHz) δ 8.09 (t, *J* = 8.34 Hz, 2H, Ar*H*); ^13^C NMR (CDCl_3_, 100 MHz) δ 113.97 (dd, *J* = 14.25, 7.66 Hz, 2 × C), 125.19 (t, *J* = 5.23 Hz, 2 × C), 153.50 (d, *J* = 16.46 Hz), 153.68 153.50 (2 × C), 156.15 (d, *J* = 16.39 Hz); IR (KBr): 3143, 3068, 2836, 2782, 2718, 2611, 1625, 1571, 1539, 1511, 1389, 1218, 1161, 1104, 893, 814 cm^−1^; EIMS *m/z*: 238 (M^+^ + 4, 11), 236 (M^+^ + 2, 78), 234 (M^+^, 100), 234 (30), 218 (12), 216 (36), 208 (24), 206 (38), 171 (27), 164 (30), 159 (18), 138 (11), 136 (16), 124 (10), 88 (15), 75 (11); HRMS calcd. For C_8_H_2_Cl_2_F_2_N_2_: 233.9563; found: 233.9568.

*1,4,6,7-Tetrachlorophthalazine* (**13**): Light orange solid; 84% yield; mp 135–136 °C; ^1^H NMR (CDCl_3_, 400 MHz) δ 8.40 (s, 2H, Ar*H*); ^13^C NMR (CDCl_3_, 100 MHz) δ 126.07 (2 × C), 127.39 (2 × C), 140.2 (2 × C), 153.45 (2 × C); IR (KBr): 3094, 1596, 1535, 1453, 1501, 1380, 1268, 1242, 1130, 1035, 702, 663 cm^−1^; EIMS *m/z*: 270 (M^+^ + 2, 26), 270 (M^+^ + 2, 16), 268 (M^+^, 100), 266 (77), 240 (19), 238 (14), 205 (14), 203 (14), 196 (13), 84 (10); HRMS calcd. For C_8_H_2_Cl_4_N_2_: 265.8972; found: 265.8979.

*6,7-Dichloropyrazino[2,3-d] pyridazine* (**14**) [28]: Black solid; 31% yield; mp 204–206 °C; ^1^H NMR (CDCl_3_, 400 MHz) δ 9.14 (s, 1H, Ar*H*), 9.17 (s, 1H, Ar*H*) [28].

*1,4-Dichlorobenzo[g]phthalazine* (**15**): Gray solid; 85% yield; mp 212–214 °C; ^1^H NMR (CDCl_3_, 400 MHz) δ 7.78–7.81 (m, 2H, Ar*H*), 8.18–8.21 (m, 2H, Ar*H*), 8.82 (s, 2H, Ar*H*); ^13^C NMR (CDCl_3_, 100 MHz) δ 123.23 (2 × C), 126.83 (2 × C), 129.22 (2 × C), 129.79 (2 × C), 135.46 (2 × C), 155.39 (2 × C); IR (KBr): 2961, 2932, 2857, 1739,1725, 1461, 1282, 1264, 1121, 1075, 739 cm^−1^; EIMS *m/z*: 250 (M^+^ + 2, 59), 249 (M^+^ + 1, 11), 248 (M^+^, 100), 178 (31), 152 (19), 151 (14), 150 (18); HRMS calcd. For C_23_H_18_N_6_O_2_: 247.9908; found: 247.9906.

## 4. Conclusions

*N*-aminophthalimides **2** and phthalazine 1,4-diones **3** were successfully and selectively synthesized from isobenzo-furan-1,3-diones (**1a**–**c**), isoindoline-1,3-dione (**1d**), furo[3,4-*b*] pyrazine-5,7-dione (**1e**), and 1*H*-pyrrolo[3,4-*c*] pyridine-1,3-dione (**1f**) with monohydrate hydrazine under the different reaction condition. The structural divergence between *N*-aminophthalimides **2f** and phthalazine 1,4-diones **3f** was effectively identified via Vilsmeier reaction methodology. Furthermore, the thermodynamically transformation from *N*-aminophthalimides **2a**–**f** to phthalazine 1,4-diones **3a–f** was successfully found, which provides the good conversion ratio from 6/94 to 1/99 of **2a**–**f** /**3a**–**f** under acetic acid mediated solution.

## Figures and Tables

**Table 1:** The thermodynamic-kinetic control synthesis of *N*-aminophthalimides **2a**–**f** and phthalazine 1,4-diones **3a**–**f**.

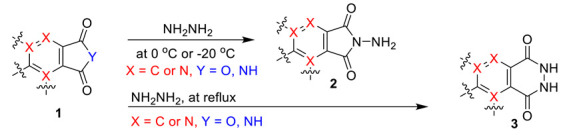		
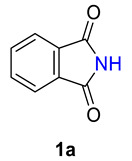	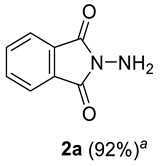	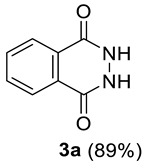
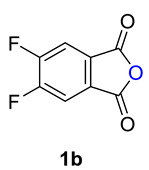	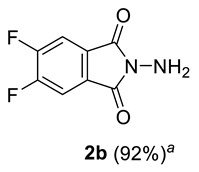	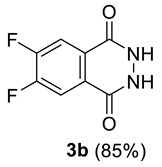
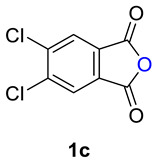	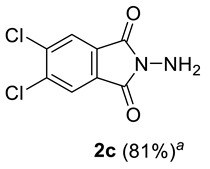	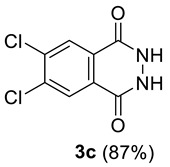
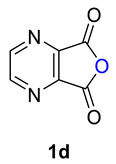	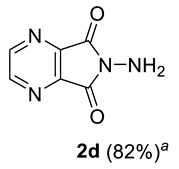	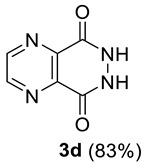
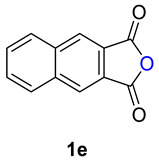	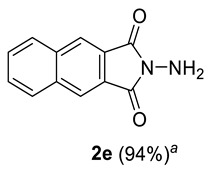	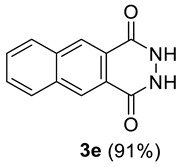
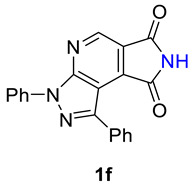	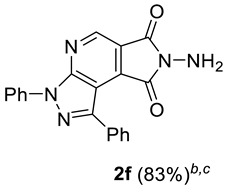	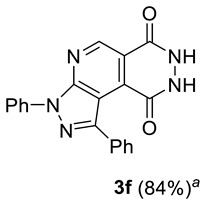

*^a^* The reaction condition at –20 ^°^C within 4h. *^b^* The reaction condition at 0 °C within 4h. *^c^* Compound **2f** and **3f** were provided and prepared from our previous work [28].

**Table 2:** Derivatization results of *N*-aminophthalimides **2a**–**e** and phthalazine 1,4-diones **3a**–**e** with Vilsmeier reagent.

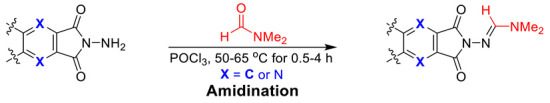
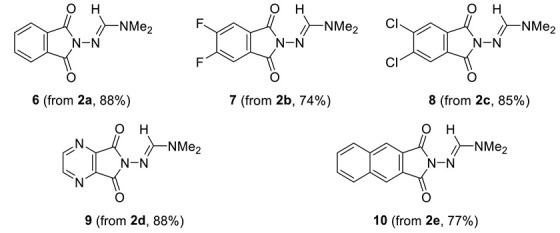
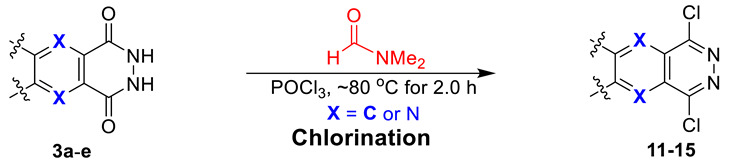
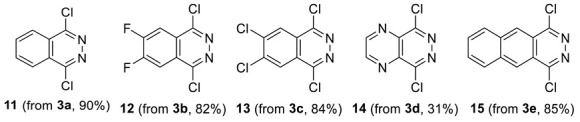

**Table 3:** Derivatization results of *N*-aminophthalimides **2a–e** and phthalazine 1,4-diones **3a–e** with Vilsmeier reagent.

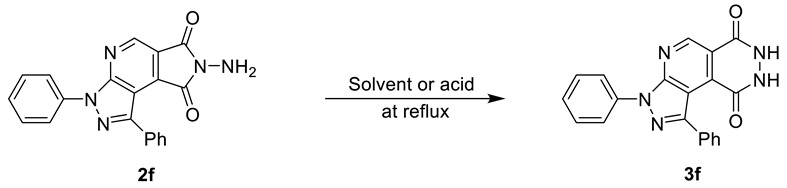					
Entry	S.M.	Solvent	Reaction Time (h)	Product	Ratio of 2f/3f ^a^
1	**2f**	CH_2_Cl_2_	24	**3f**	non-conversion
2	**2f**	Toluene	24	**3f**	non-conversion
3	**2f**	EtOH	24	**3f**	non-conversion
4	**2f**	THF	24	**3f**	88/12
5	**2f**	CH_3_CN	24	**3f**	93/7
6	**2f**	Dioxane	24	**3f**	91/9
7	**2f**	DMSO	24	**3f**	89/11
8	**2f**	TsOH (pKa = –1.9)	4	**3f**	61/39
9	**2f**	TsCl	4	**3f**	56/44
10	**2f**	TFA (pKa = 0.30)	4	**3f**	54/46
11	**2f**	AcOH (pKa = 4.76)	4	**3f**	6/94

^a^ The ratio was identified by ^1^H-NMR.

**Table 4:** The conversion results between *N*-aminophthalimide **2a**–**e** and pyrazolopyridopyridazine dione **3a**–**e**.

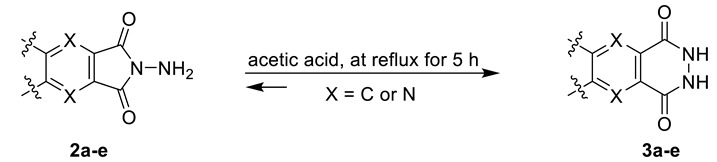				
Entry	S.M.	Reaction Time (h)	Product	2a–e/3a–e ^a^
1	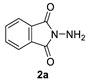	8	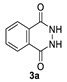	1/99
2	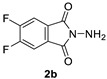	9	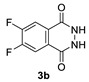	3/97
3	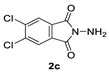	10	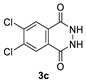	6/94
4	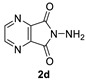	11	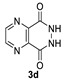	5/95
5	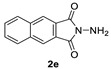	3	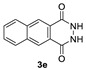	4/96

^a^ The ratio of **2**/**3** was determined by crude ^1^H-NMR.

## Data Availability

The data presented in this study are available in the article and the Supplementary Materials.

## Supplementary Materials

The following are available online, copies of ^1^H and ^13^C-NMR spectra of compounds **2a**–**2e**, **3a**–**3e**, and **4**–**15**, Table S1: Crystal data and structure refinement for *N’*-(6,8-dioxo6,8-dihydropyrazolopyrrolo-pyridine-yl)-*N*,*N*-dimethylformimidamide **4** (CCDC No. 1954819).

## Sample Availability

Samples of the compounds are available from the authors.
