# Supplementary material for: Structural Identification between Phthalazine-1,4-Diones and N-Aminophthalimides via Vilsmeier Reaction: Nitrogen Cyclization and Tautomerization Study
Source: Molecules. 2021 May 13;26(10):2907. doi: 10.3390/molecules26102907 (PMC8153572; doi:10.3390/molecules26102907)
Supplement: Supplementary file 1 [file molecules-26-02907-s001.zip › molecules-1216283-supplementary.pdf]

molecules

MDPI

Article

Structural Identification between Phthalazine-1,4-diones and *N*-Aminophthalimides via Vilsmeier Reaction: Nitrogen Cyclization and Tautomerization Study

Cheng-Yen Chung [1,2], Ching-Chun Tseng [1,2], Sin-Min Li [3], Shuo-En Tsai [1,2], Hui-Yi Lin [1,*] Fung Fuh Wong [1,*]

1 School of Pharmacy, China Medical University, No. 91, Hsueh-Shih Rd., Taichung 40402, Taiwan; u109308001@cmu.edu.tw (C.-Y.C.); u106308201@cmu.edu.tw (C.-C.T.); u100003044@cmu.edu.tw (S.-E.T.)
2 The Ph.D. Program for Biotech Pharmaceutical Industry, China Medical University, No. 91, Hsueh-Shih Rd., Taichung, 40402, Taiwan
3 Institute of New Drug Development, China Medical University, No. 91 Hsueh-Shih Rd., Taichung 40402, Taiwan; u102003483@cmu.edu.tw (S.-M.L.)
* Correspondence: wongfungfuh@yahoo.com.tw or ffwong@mail.cmu.edu.tw; Tel.: +886-422-053-366 (ext. 5603); Fax: +886-422-078-083

Supporting information

Table of Contents

1. General Experimental Procedures....................S2

2. NMR Spectra of Reported Compounds....................S6

3. X-ray crystallography data section....................S27

2a
N-Aminophthalimide

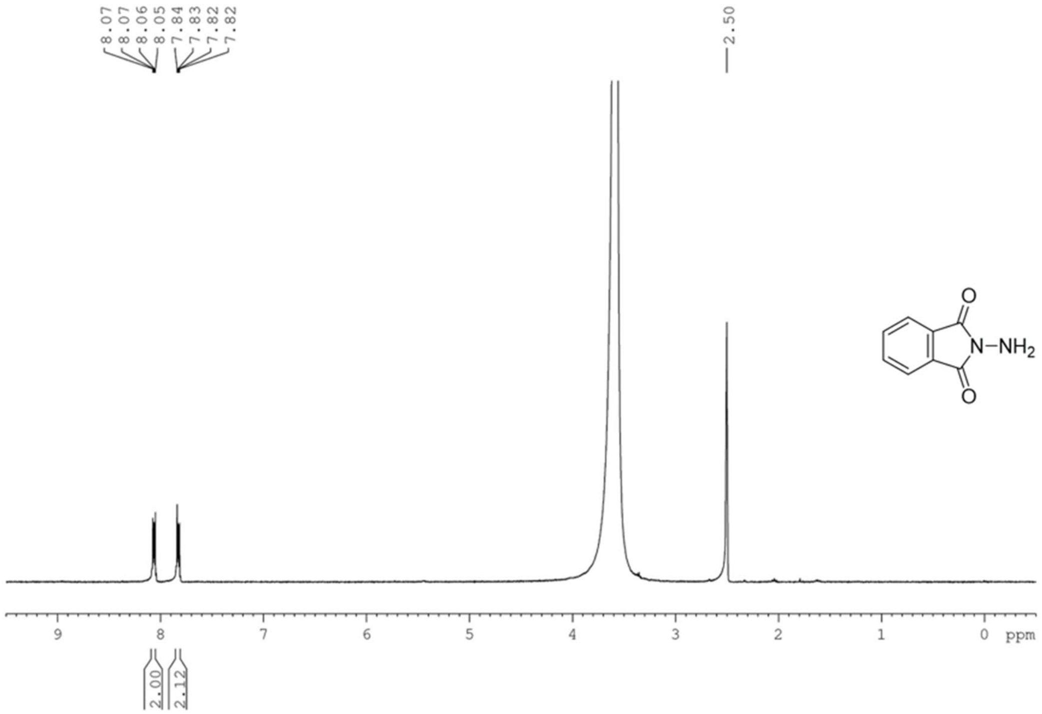

2a
N-Aminophthalimide

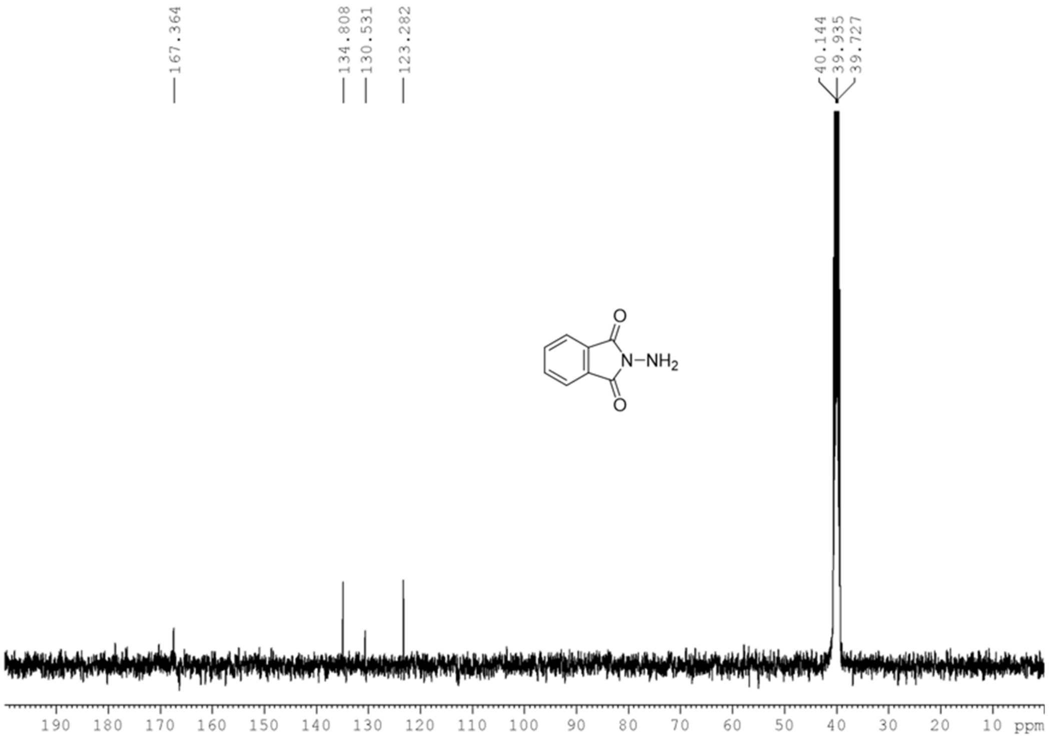

2b
N-Amino-4,5-difluoropthalimide

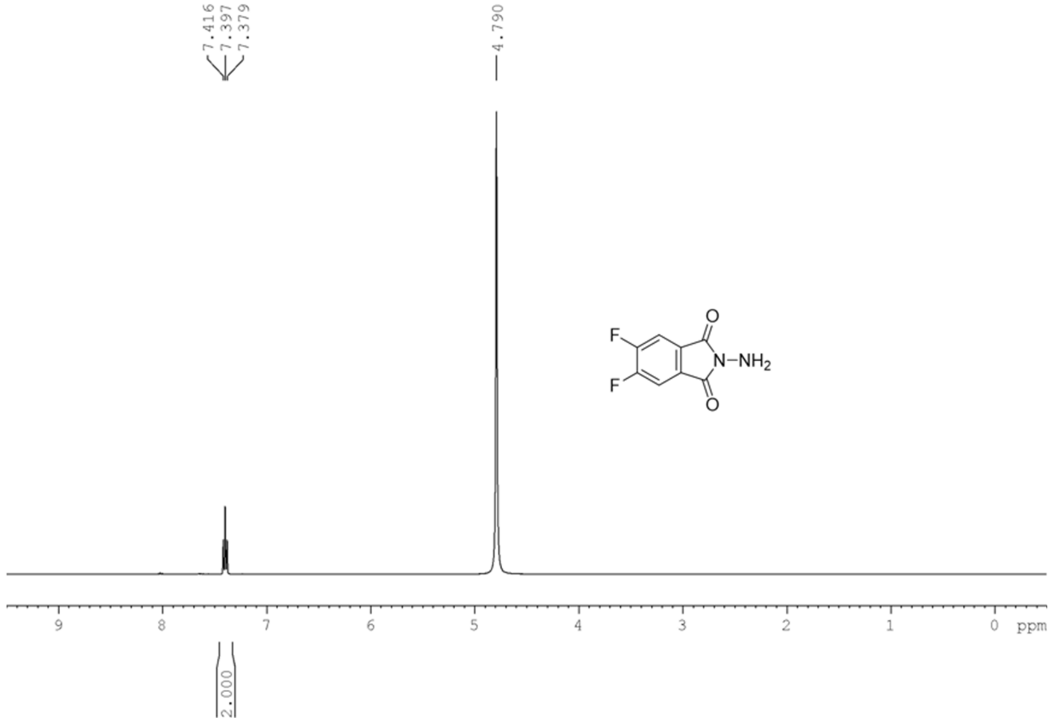

2b
N-Amino-4,5-difluoropthalimide

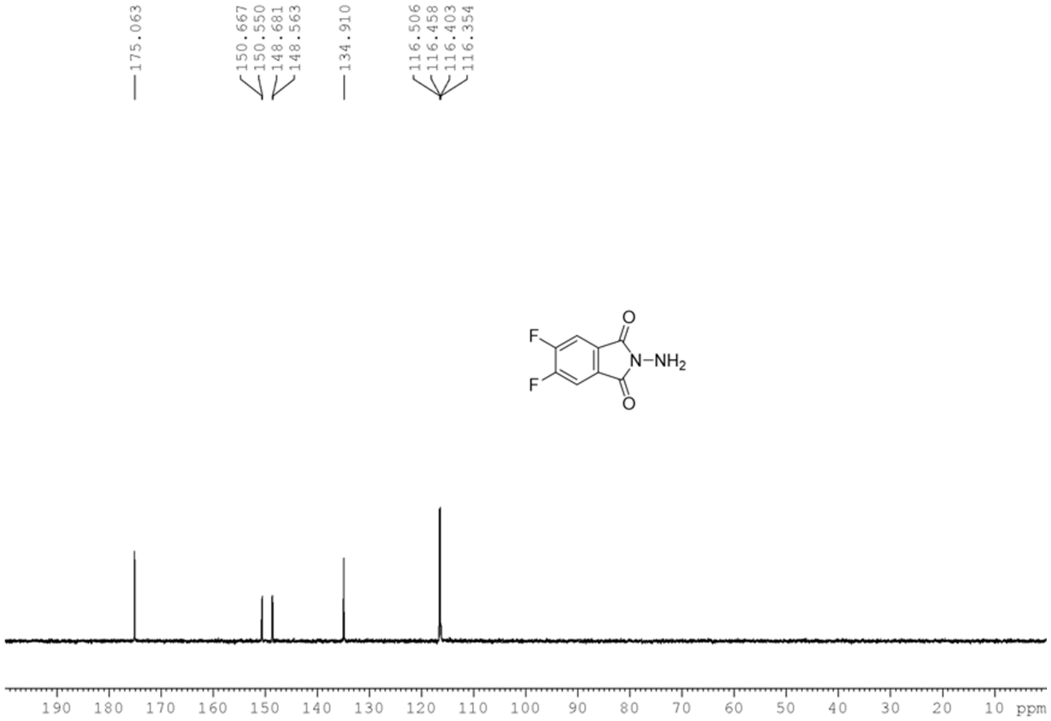

2c

N-Amino-4,5-dichlorophthalimide

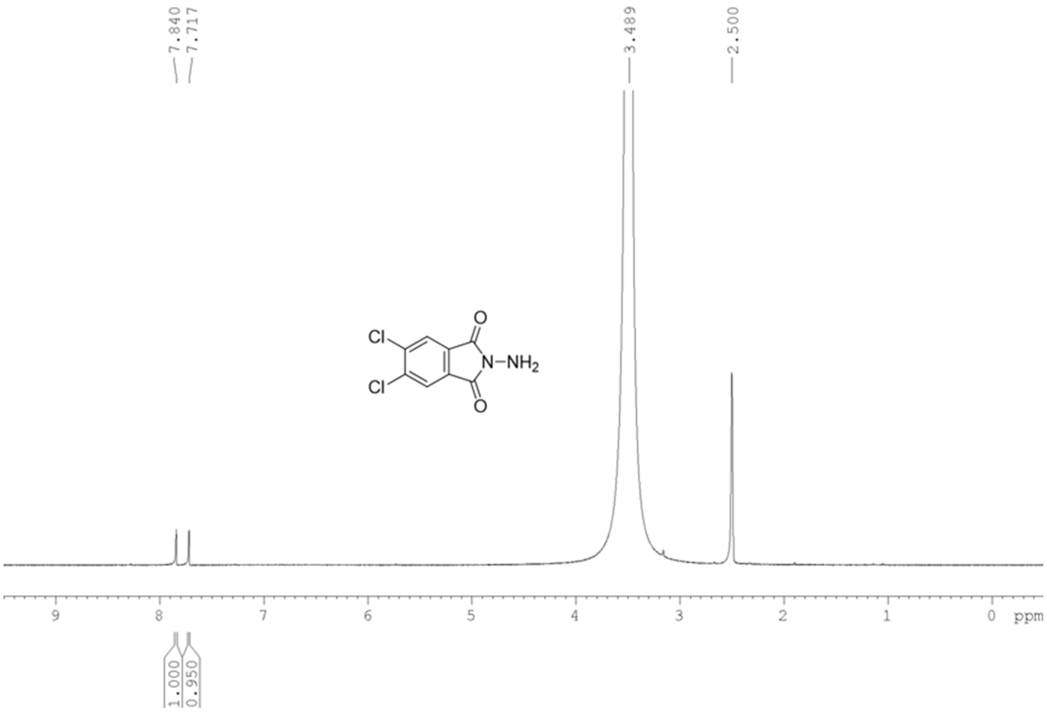

2c

N-Amino-4,5-dichlorophthalimide

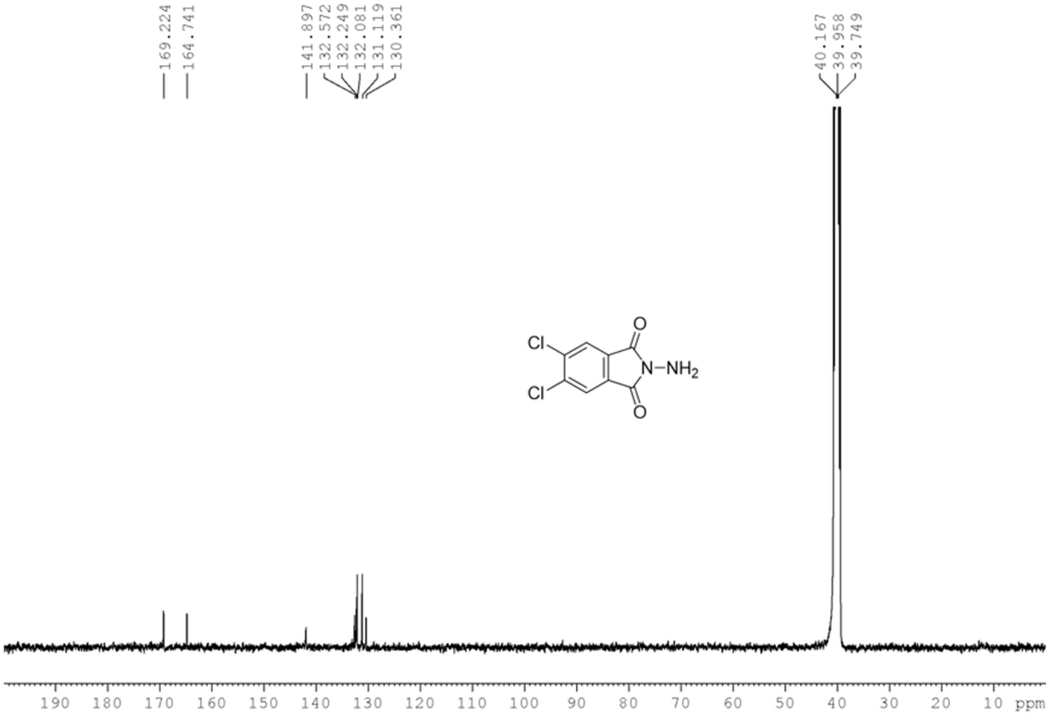

2d

N-Amino-2,3-pyrazinedicarboxylicphthalimide

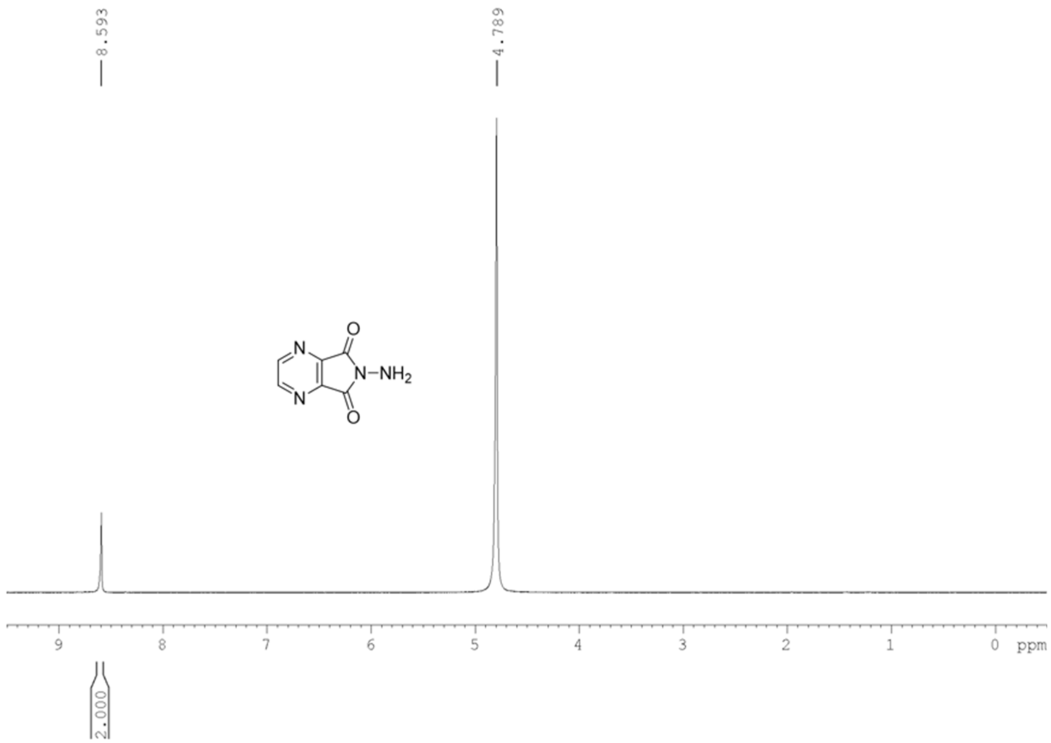

2d

N-Amino-2,3-pyrazinedicarboxylicphthalimide

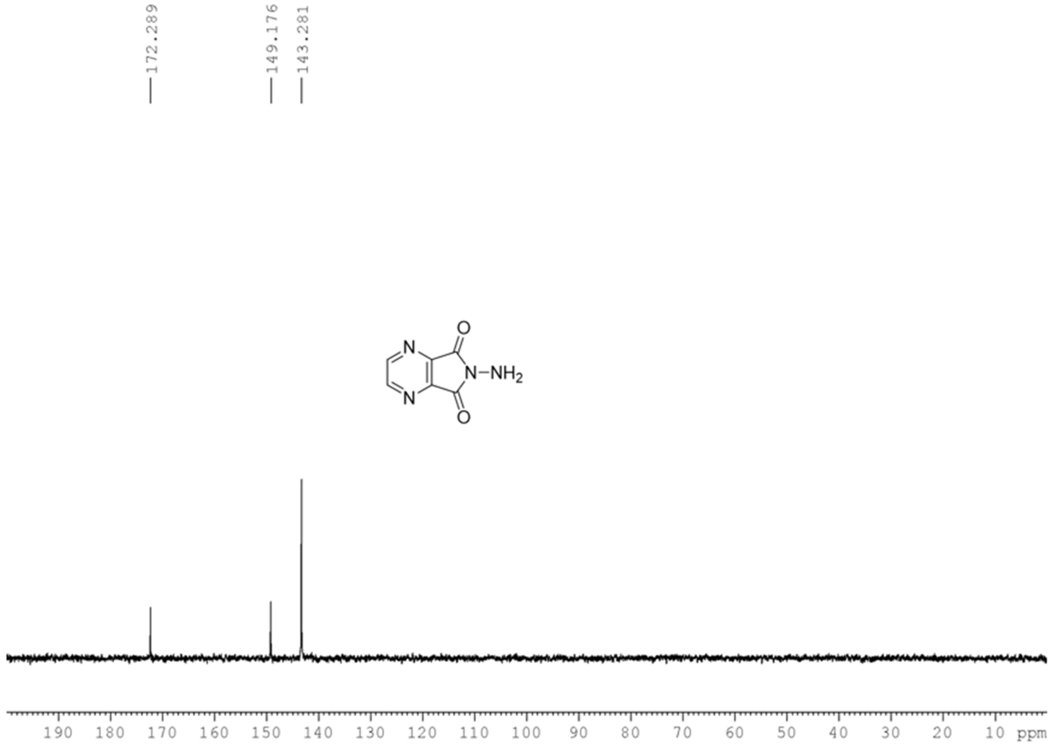

2e

Naphthalene-2,3-dicarboxylic hydrazide

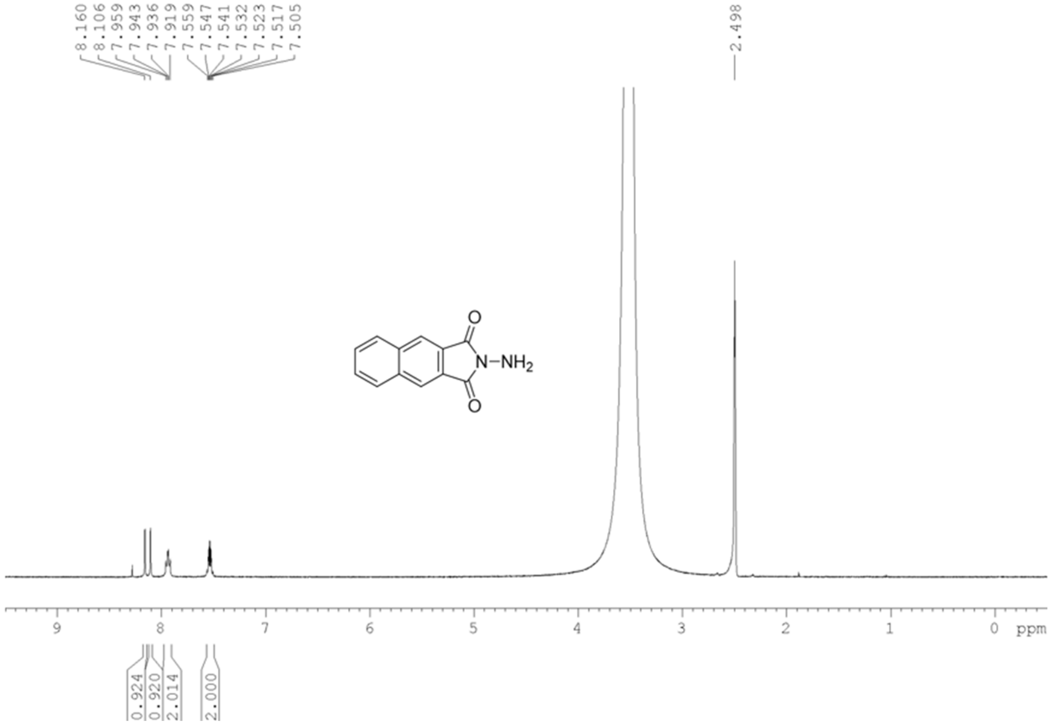

2e

Naphthalene-2,3-dicarboxylic hydrazide

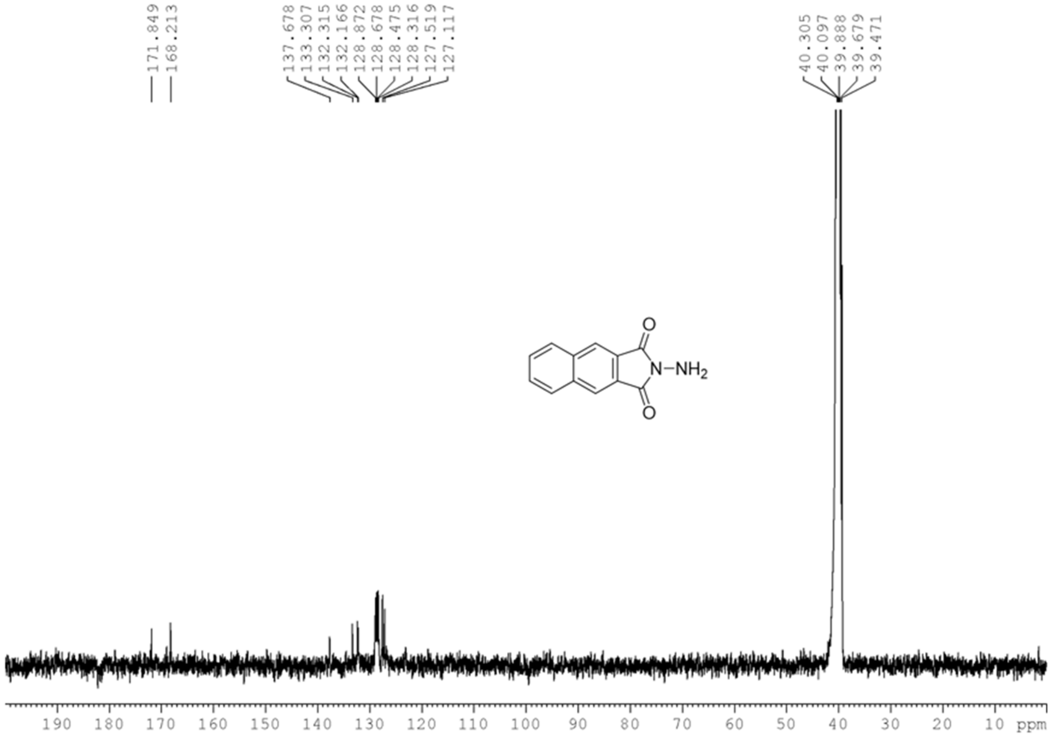

3a

2,3-Dihydro-phthalazine-1,4-dione

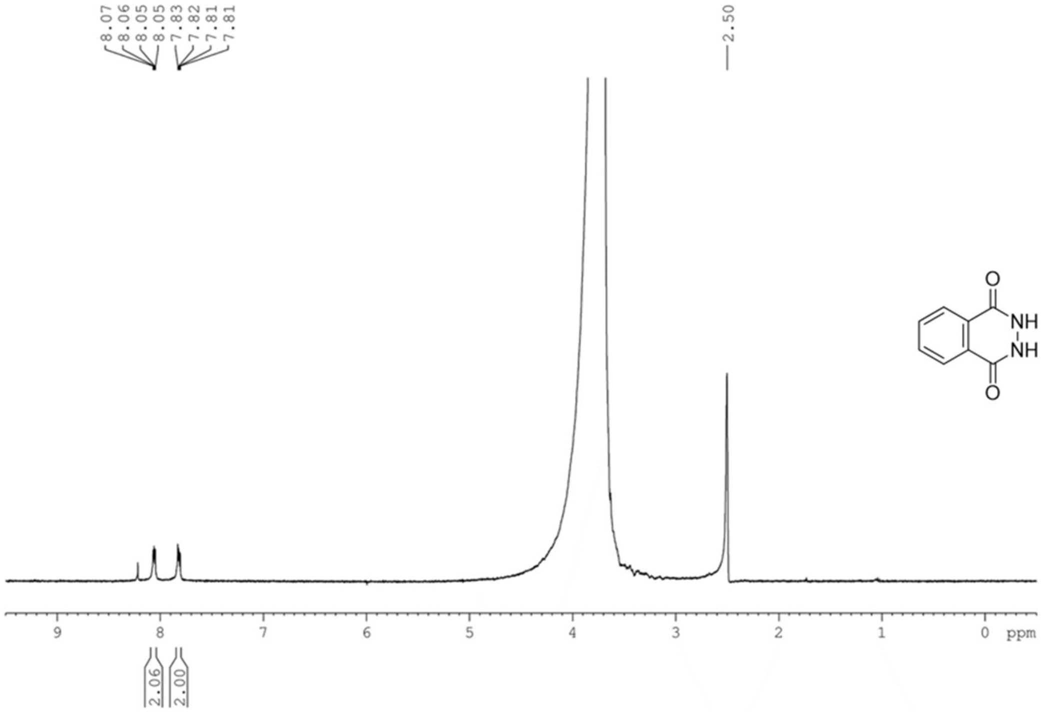

3a

2,3-Dihydro-phthalazine-1,4-dione

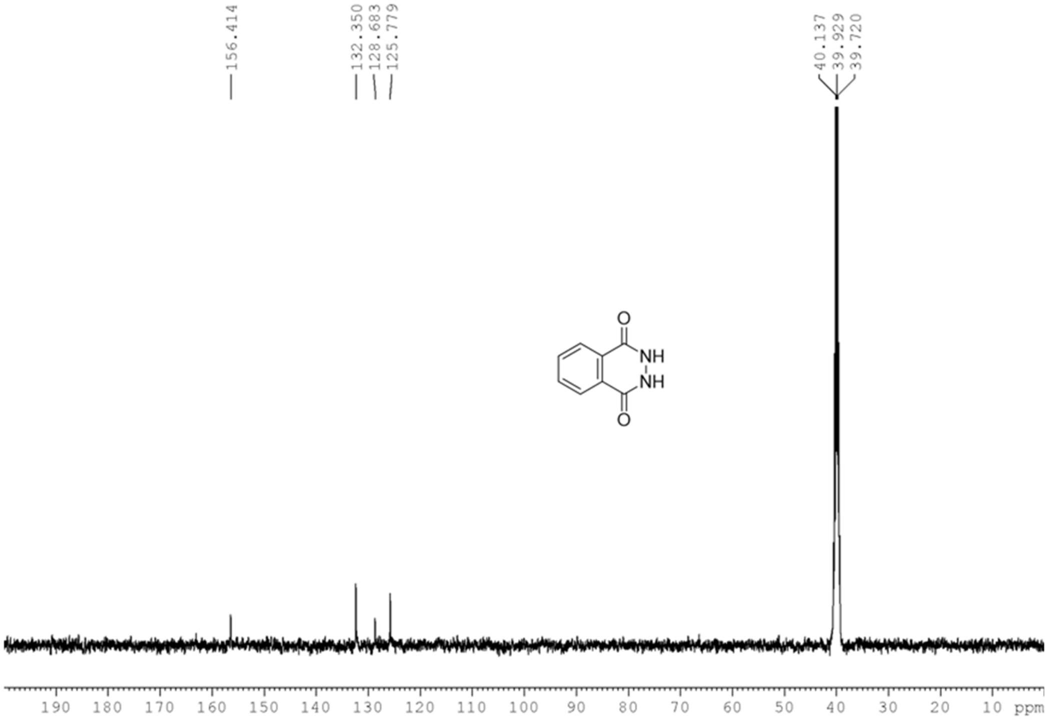

3b
6,7-Fluoroo-2,3-dihydrophthalazine-1,4-dione

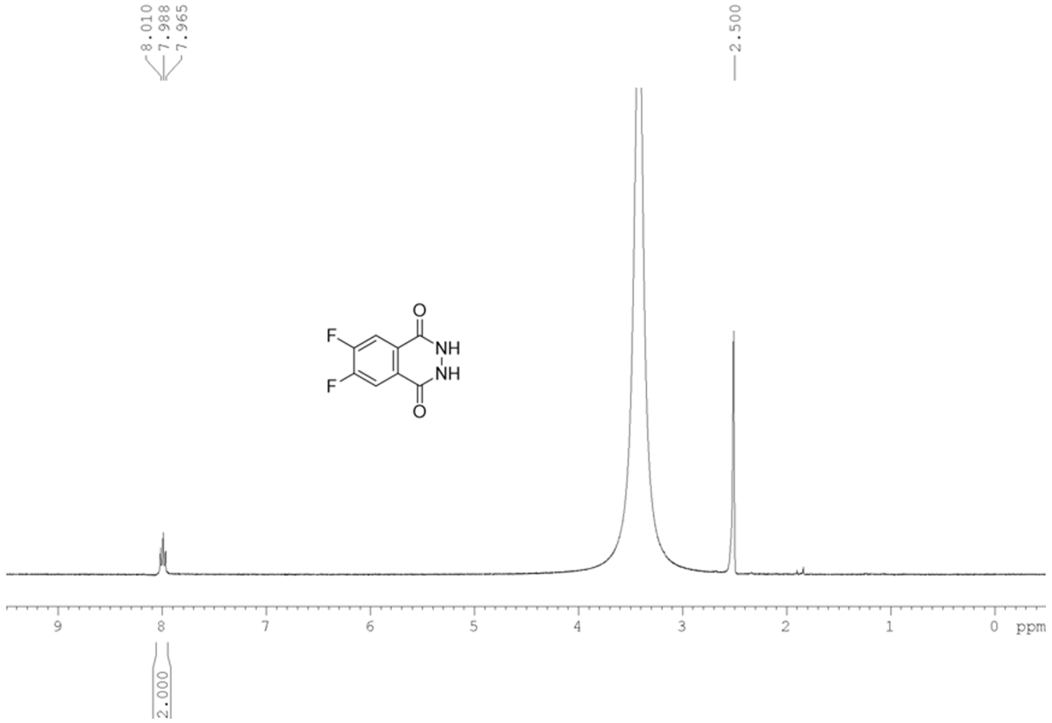

3b
6,7-Fluoroo-2,3-dihydrophthalazine-1,4-dione

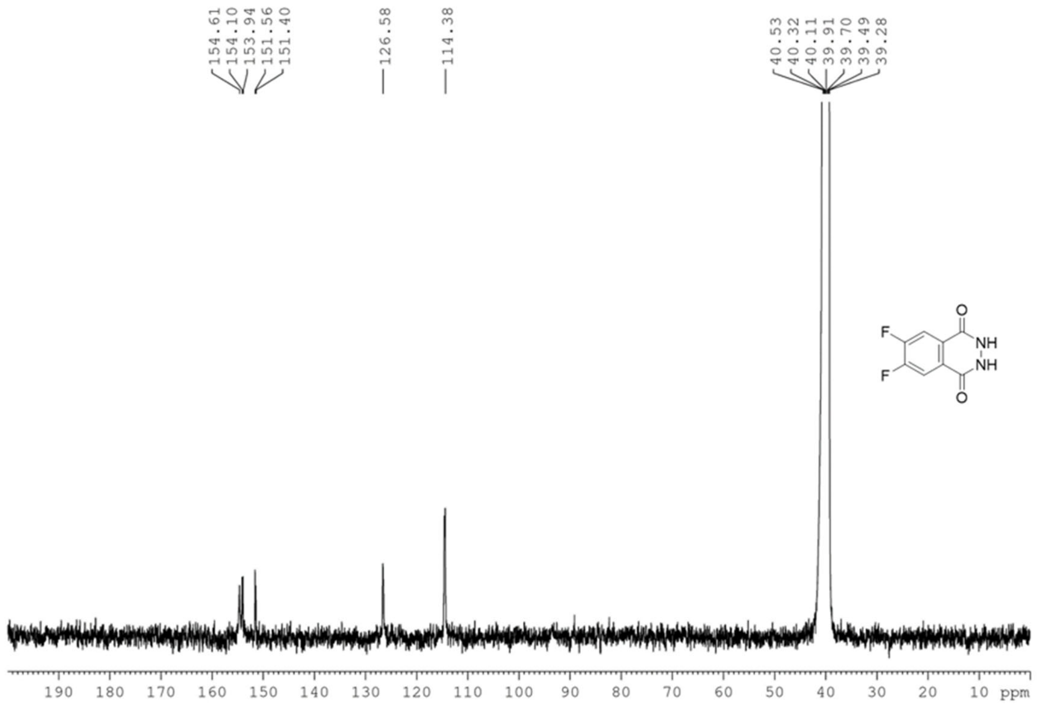

3c

6,7-Dichloro-2,3-dihydrophthalazine-1,4-dione

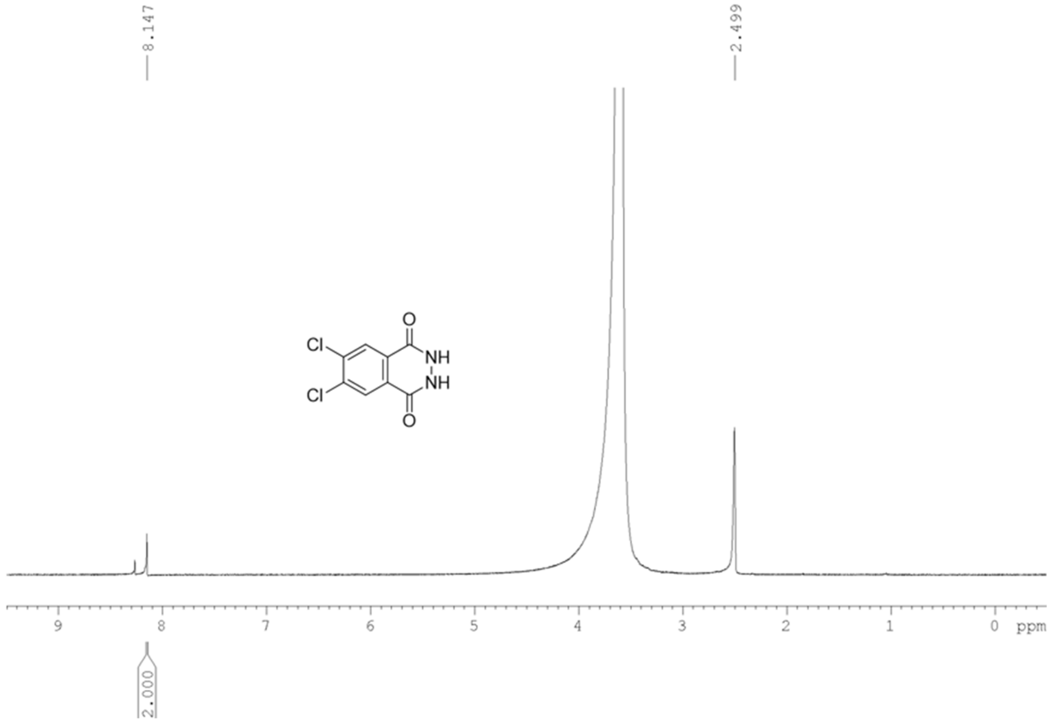

3c

6,7-Dichloro-2,3-dihydrophthalazine-1,4-dione

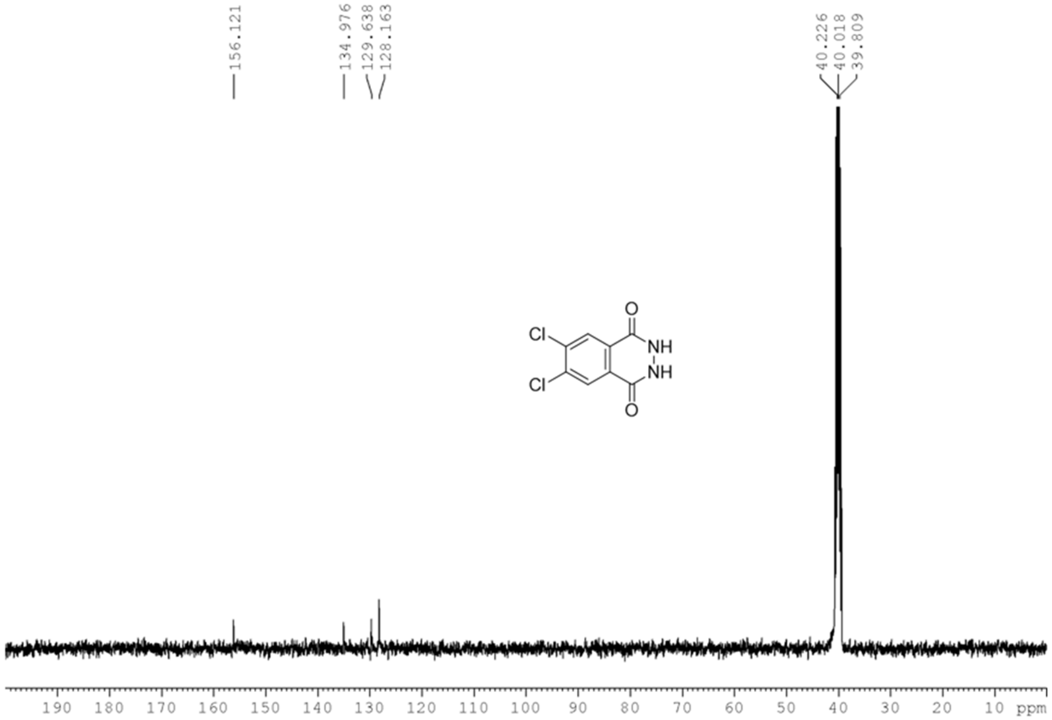

3d

7-Dihydropyrazino[2,3-*d*]pyridazine-5,8-dione

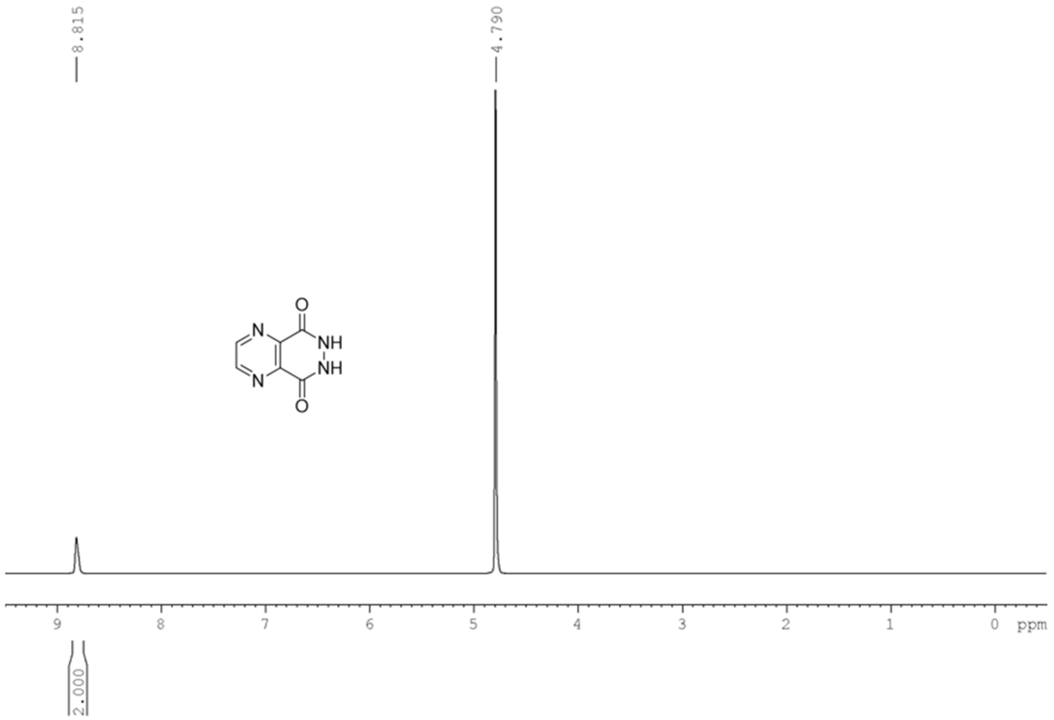

3d

7-Dihydropyrazino[2,3-*d*]pyridazine-5,8-dione

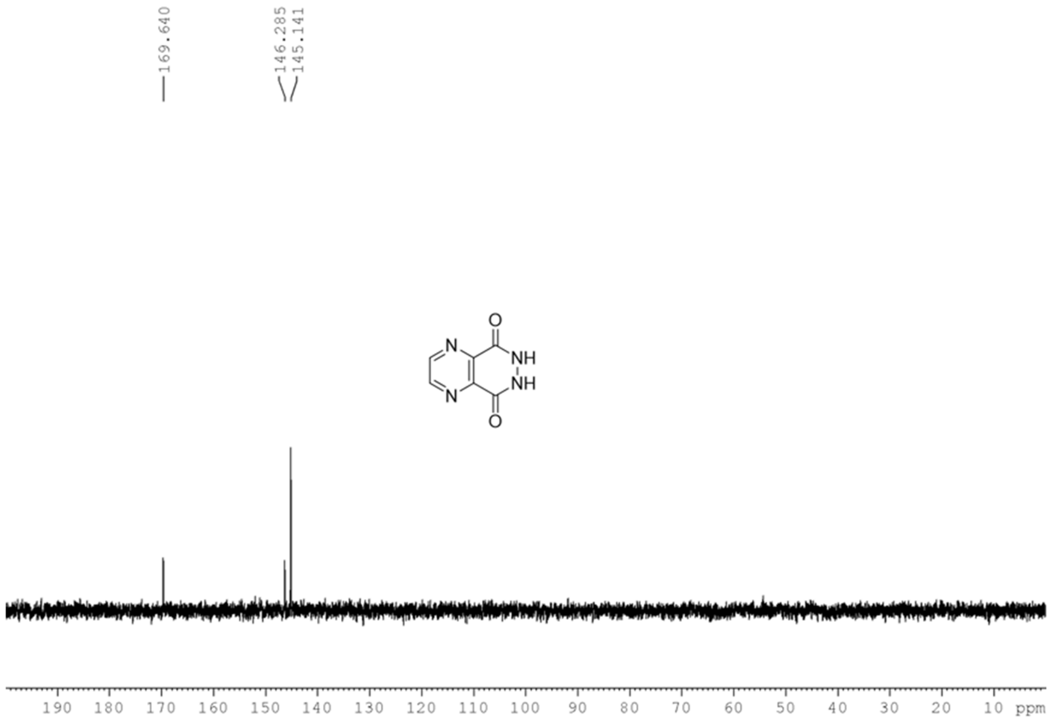

3e

2,3-Dihydrobenzo[*g*]phthalazine-1,4-dione

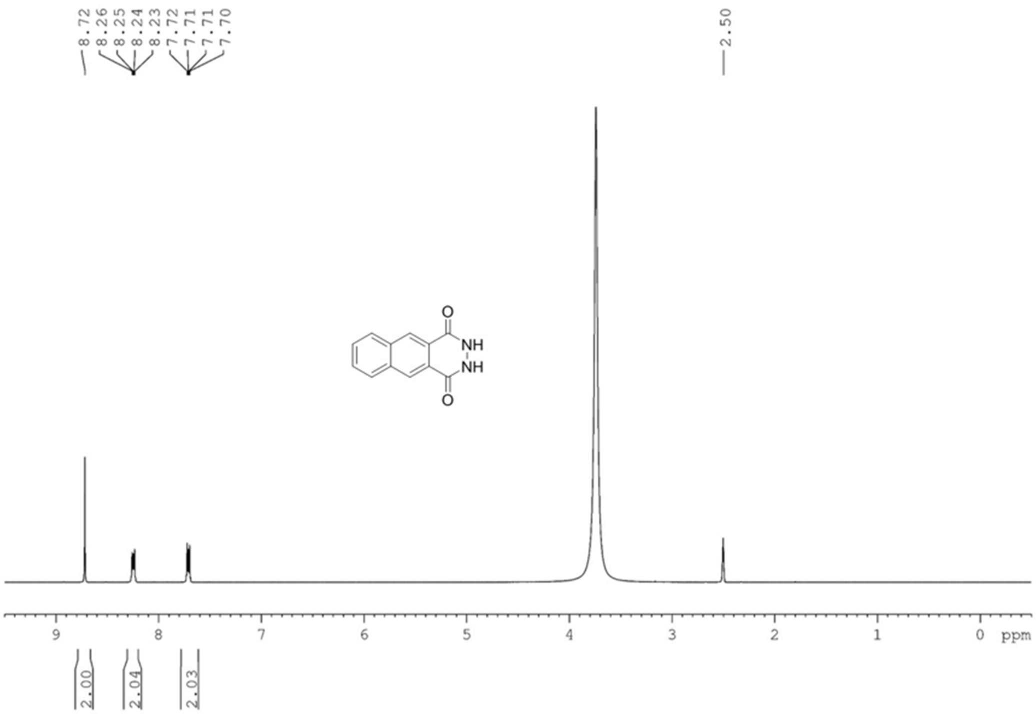

3e

2,3-Dihydrobenzo[*g*]phthalazine-1,4-dione

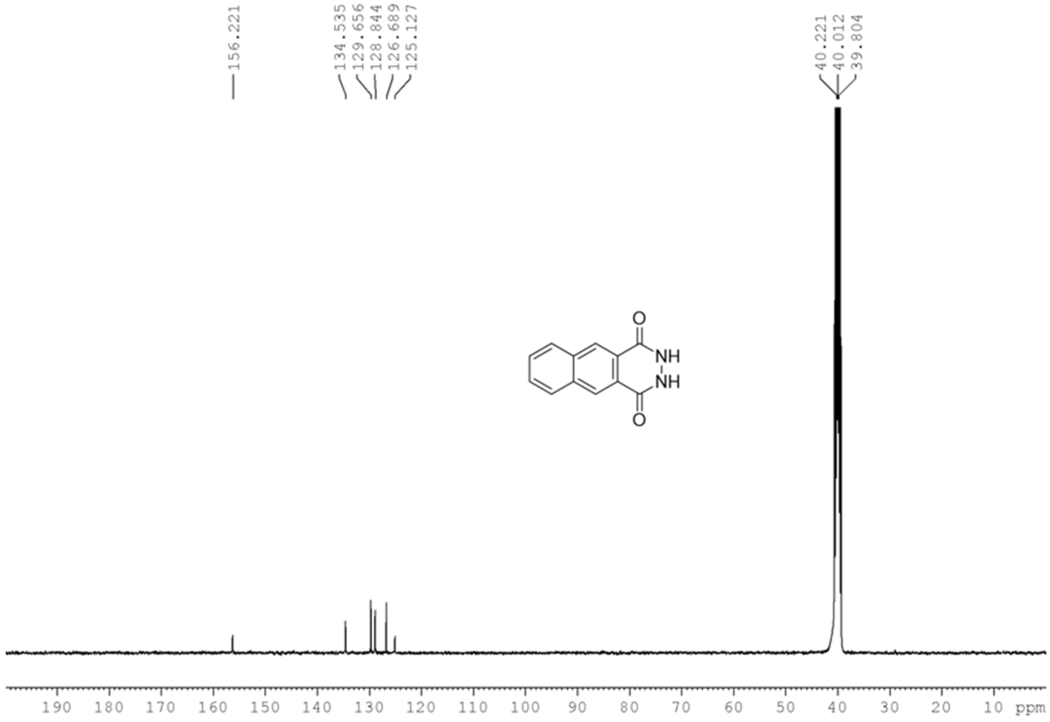

4

N'-(6,8-Dioxo-1,3-diphenyl-6,8-dihydropyrazolo[3,4-*b*]pyrrolo[3,4-d]pyridin-7(3*H*)-yl)-*N*,*N*-dimethylformimidamide

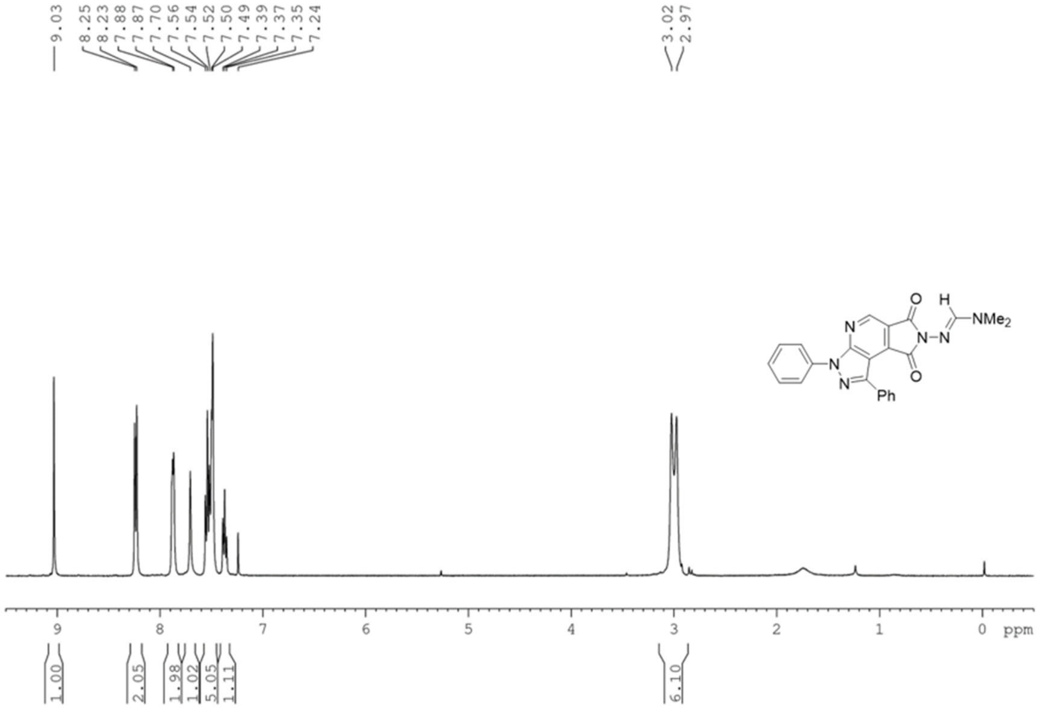

4

N'-(6,8-Dioxo-1,3-diphenyl-6,8-dihydropyrazolo[3,4-*b*]pyrrolo[3,4-d]pyridin-7(3*H*)-yl)-*N*,*N*-dimethylformimidamide

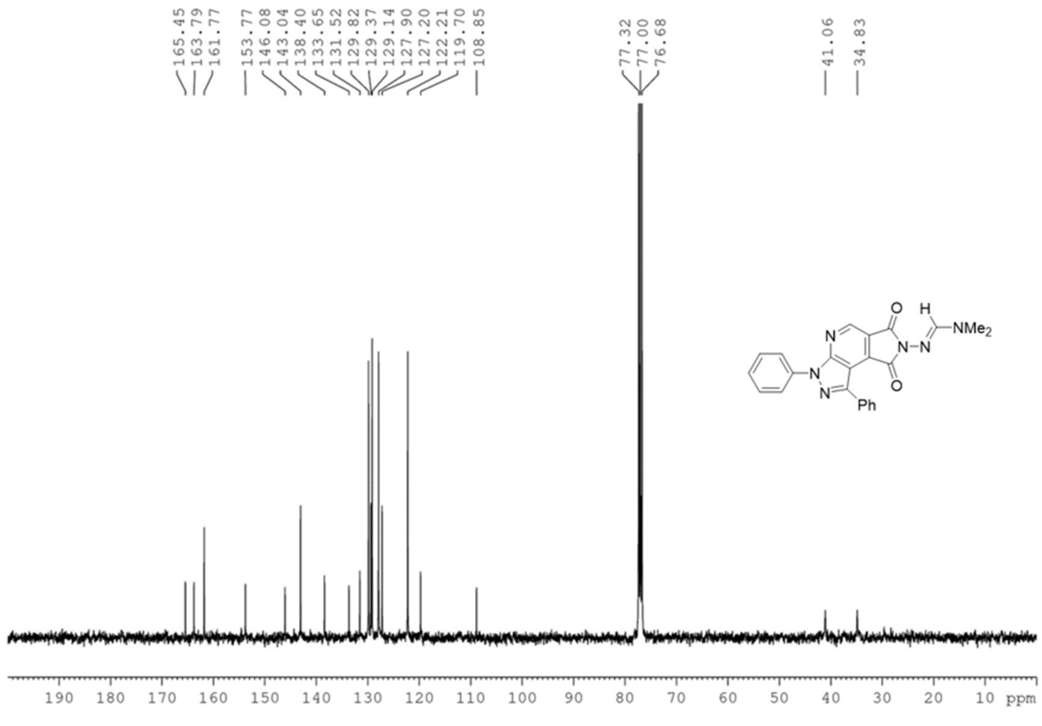

6

N'-(1,3-Dioxo-1,3-dihydro-2*H*-isoindol-2-yl)-*N*,*N*-dimethyliminoformamide hydrochloride

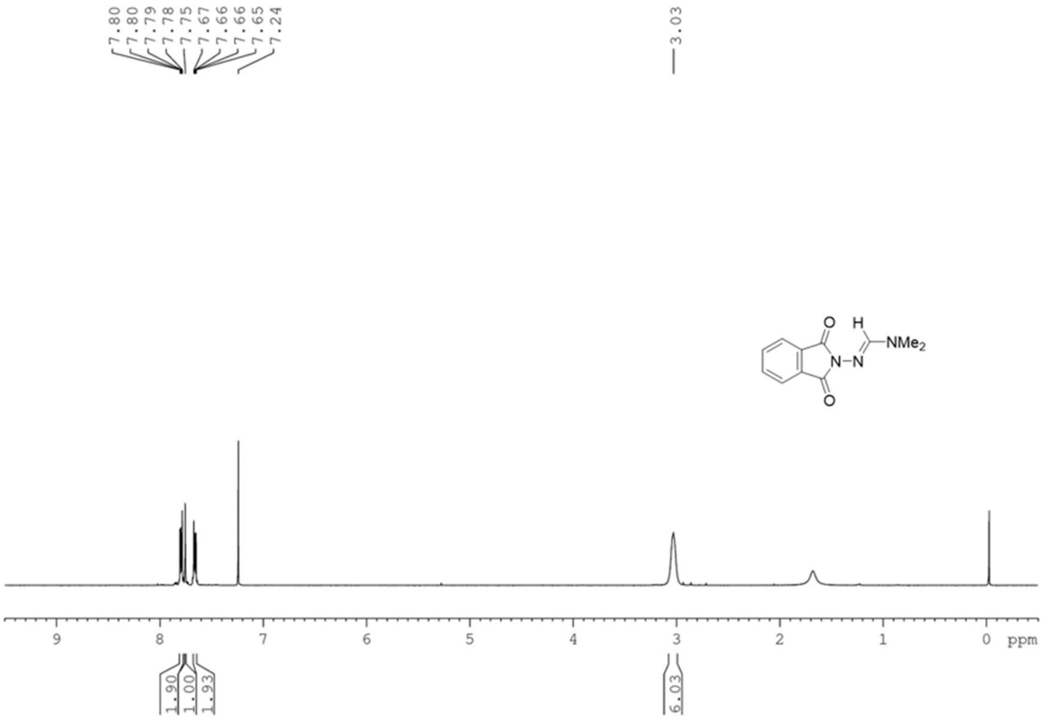

6

N'-(1,3-Dioxo-1,3-dihydro-2*H*-isoindol-2-yl)-*N*,*N*-dimethyliminoformamide hydrochloride

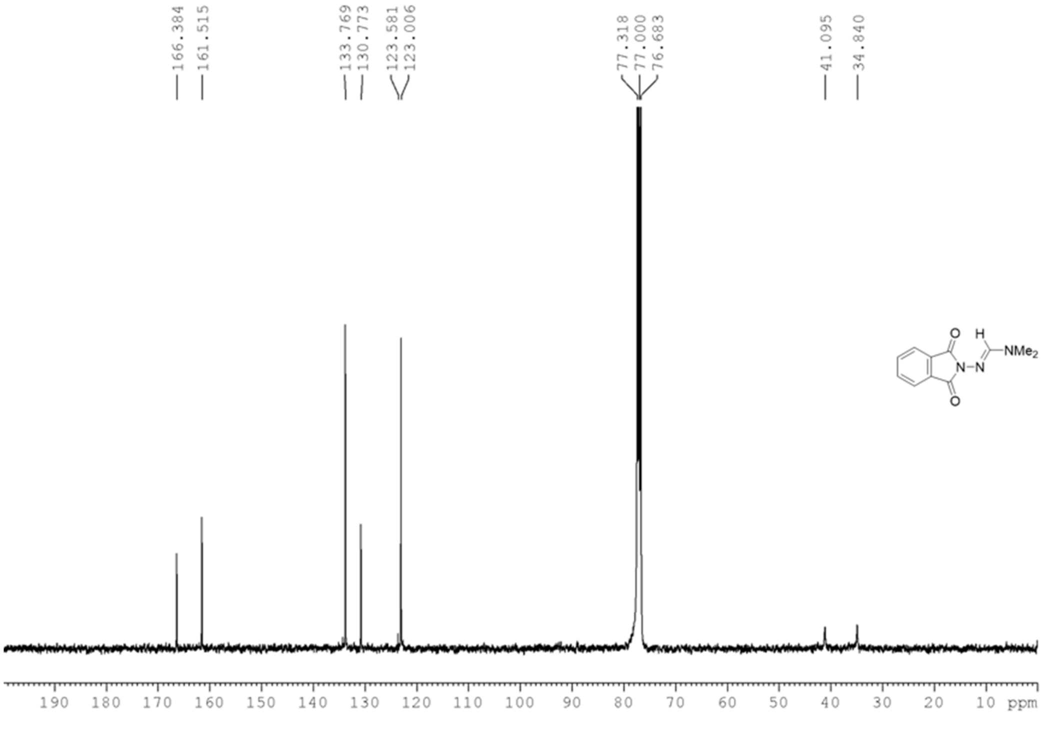

7

N'-(1,3-Dioxo-5,6-difluoro-2*H*-isoindolin-2-yl)-*N*,*N*-dimethylformimidamide

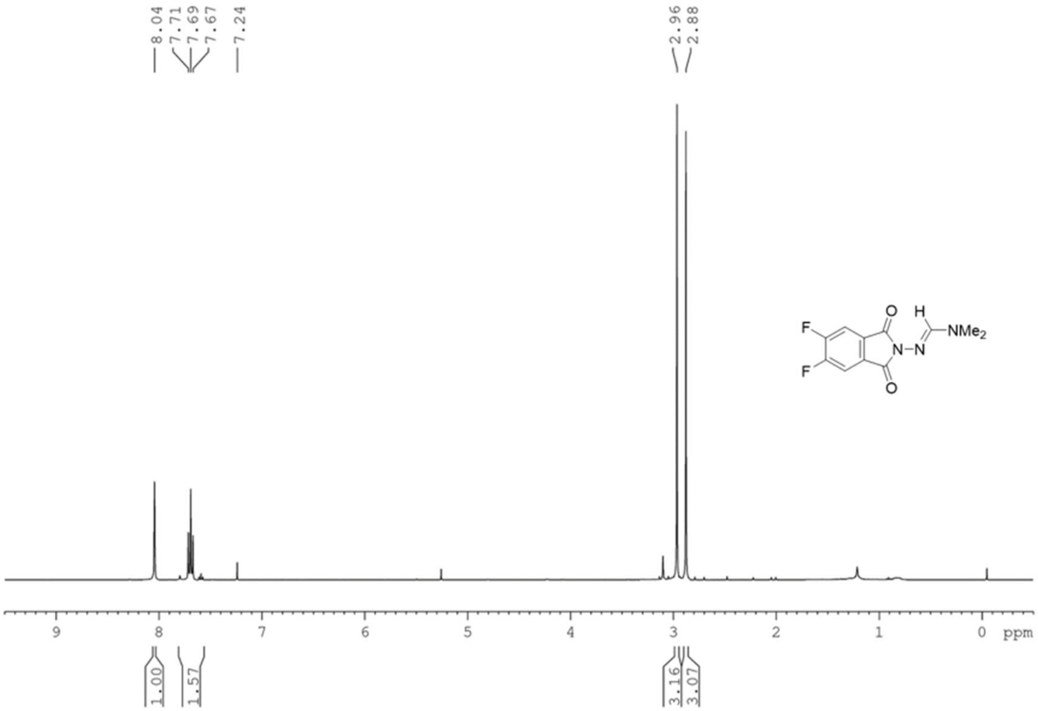

7

N'-(1,3-Dioxo-5,6-difluoro-2*H*-isoindolin-2-yl)-*N*,*N*-dimethylformimidamide

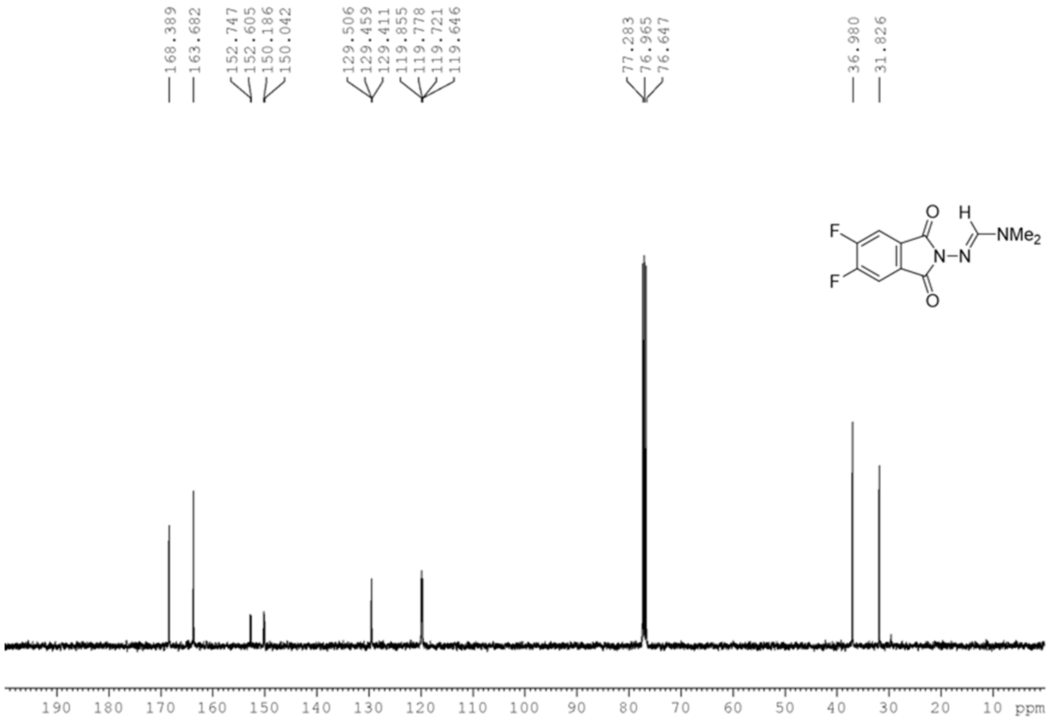

8

N'-(1,3-Dioxo-5,6-dichloro-2H-isoindolin-2-yl)-*N*,*N*-dimethylformimidamide

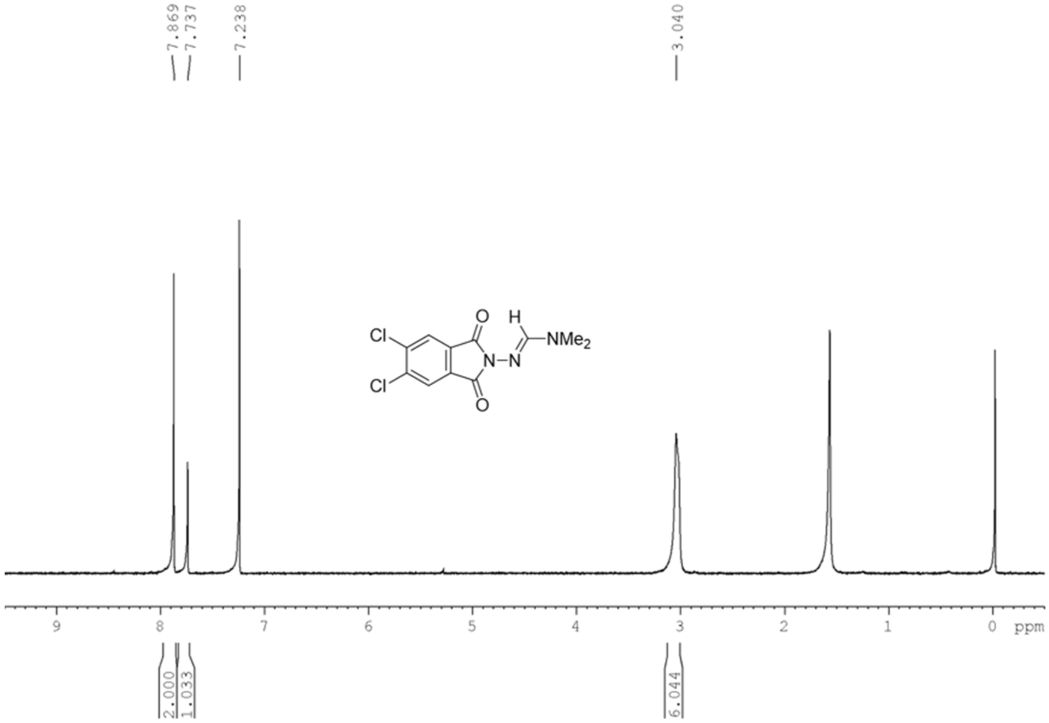

8

N'-(1,3-Dioxo-5,6-dichloro-2H-isoindolin-2-yl)-*N*,*N*-dimethylformimidamide

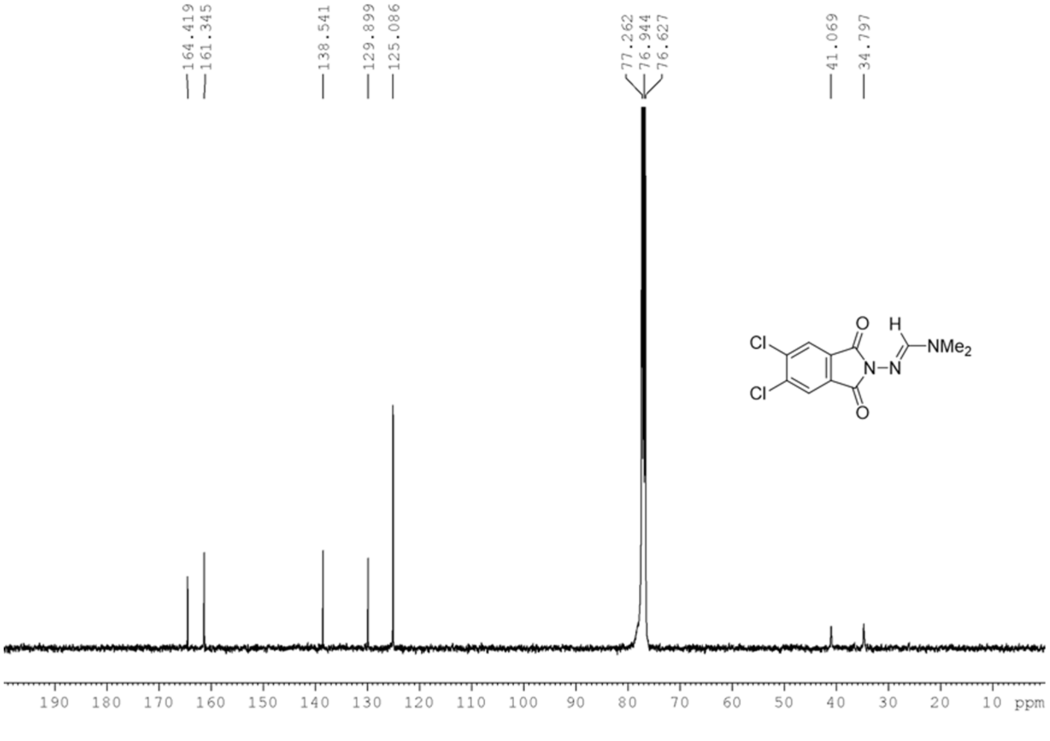

9

N'-(1,3-Dioxo-5,7-dihydro-6H-pyrrolo[3,4-*b*]pyrazin-6-yl)-*N*,*N*-dimethylformimidamide

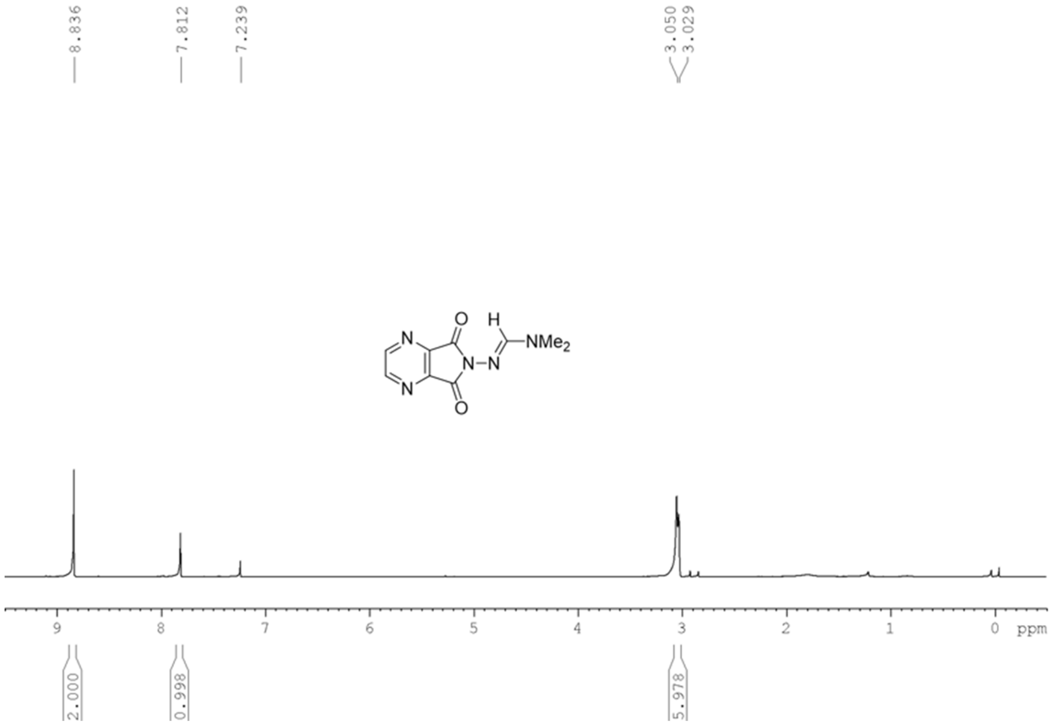

9

N'-(1,3-Dioxo-5,7-dihydro-6H-pyrrolo[3,4-*b*]pyrazin-6-yl)-*N*,*N*-dimethylformimidamide

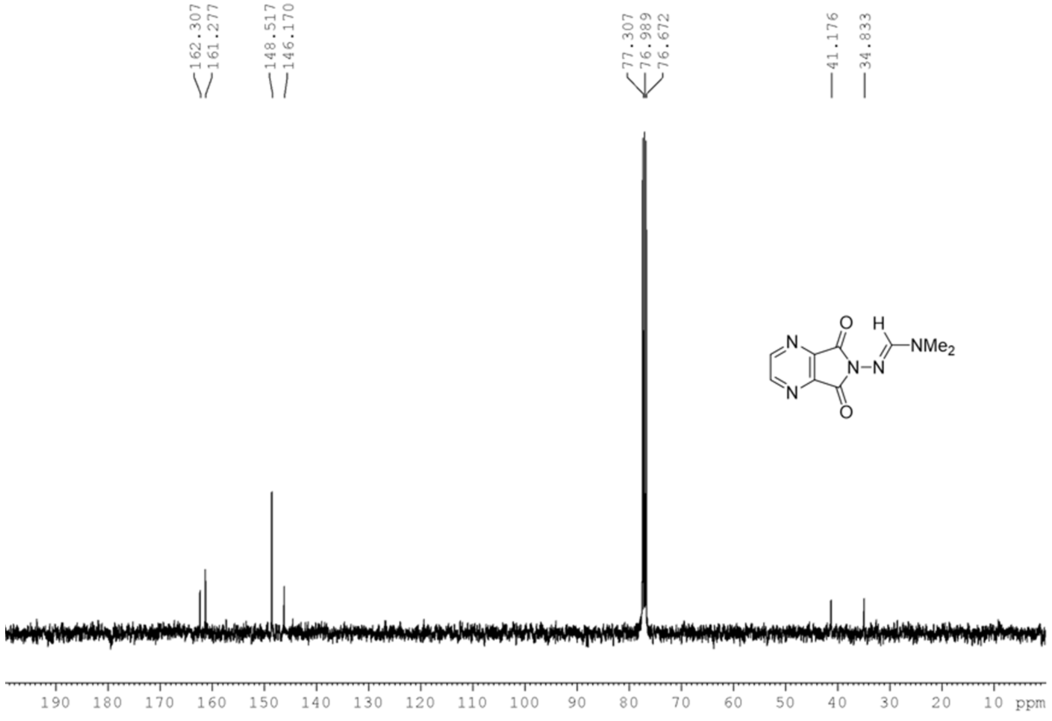

10

N'-(1,3-Dioxo-5,6-dihydro-2*H*-benzo[*f*]isoindol-2-yl)-*N*,*N*-dimethylformimidamide

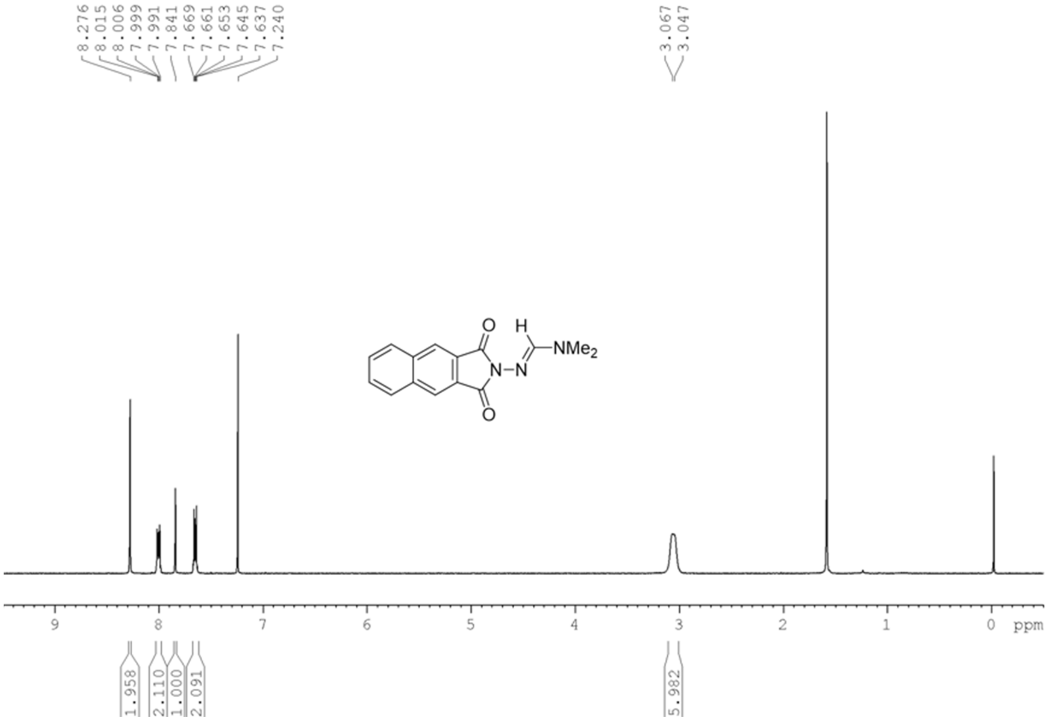

10

N'-(1,3-Dioxo-5,6-dihydro-2*H*-benzo[*f*]isoindol-2-yl)-*N*,*N*-dimethylformimidamide

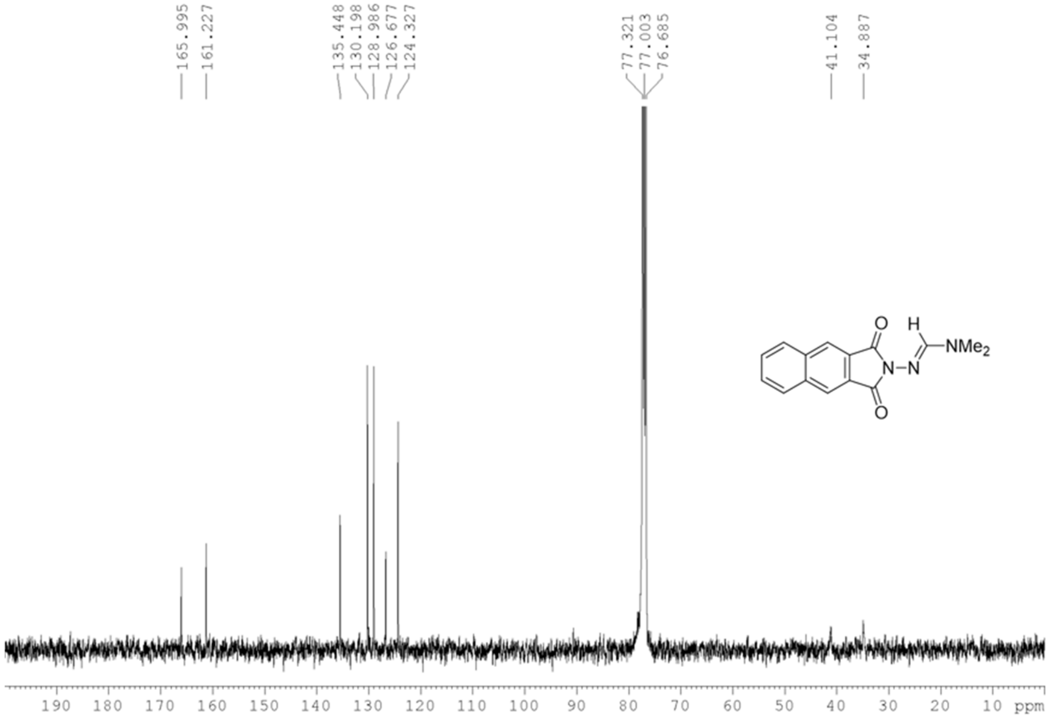

5

6,9-Dichloro-1,3-diphenyl-3*H*-pyrazolo[4',3':5,6]pyrido[3,4-*d*]pyridazine

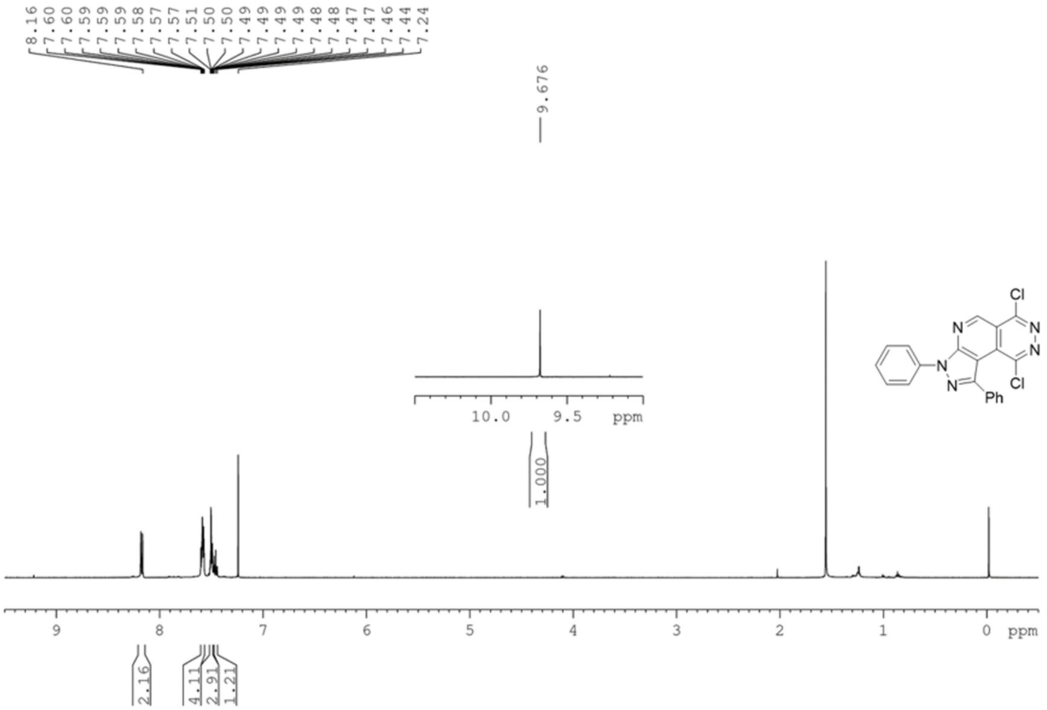

5

6,9-Dichloro-1,3-diphenyl-3*H*-pyrazolo[4',3':5,6]pyrido[3,4-*d*]pyridazine

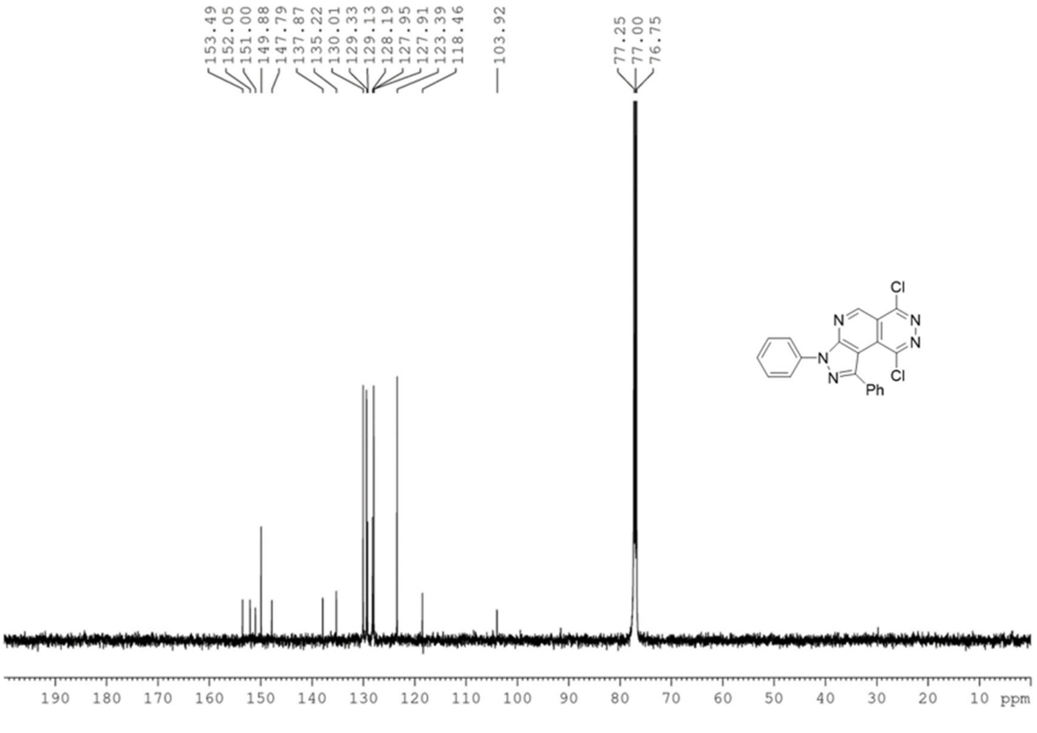

11
1,4-Dichlorophthalazine

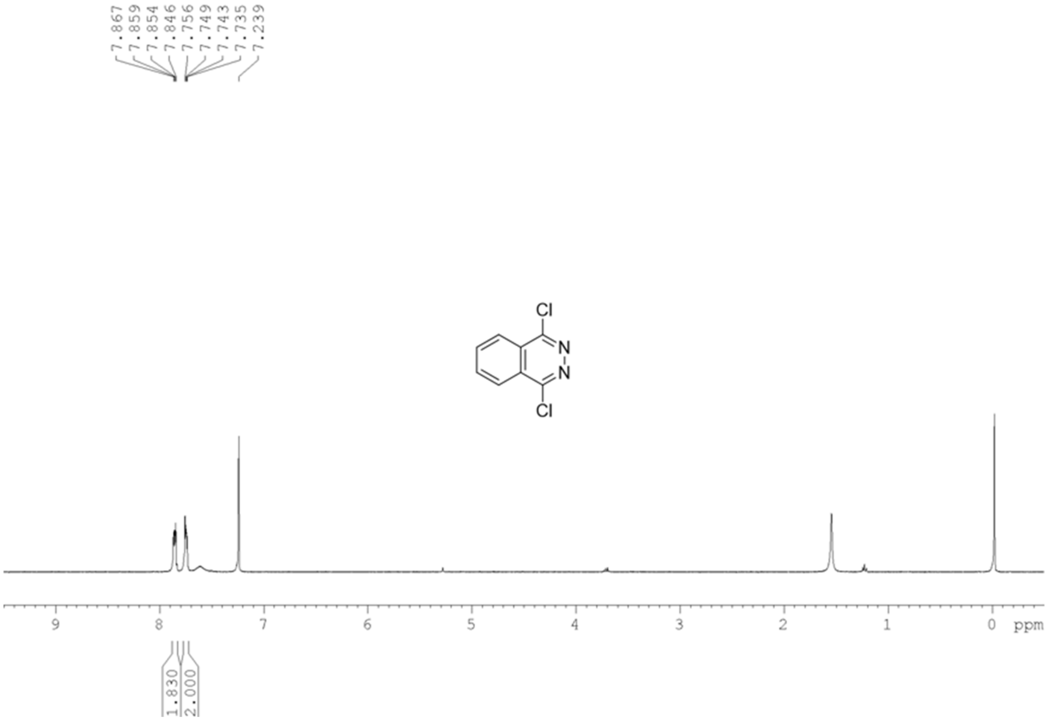

11
1,4-Dichlorophthalazine

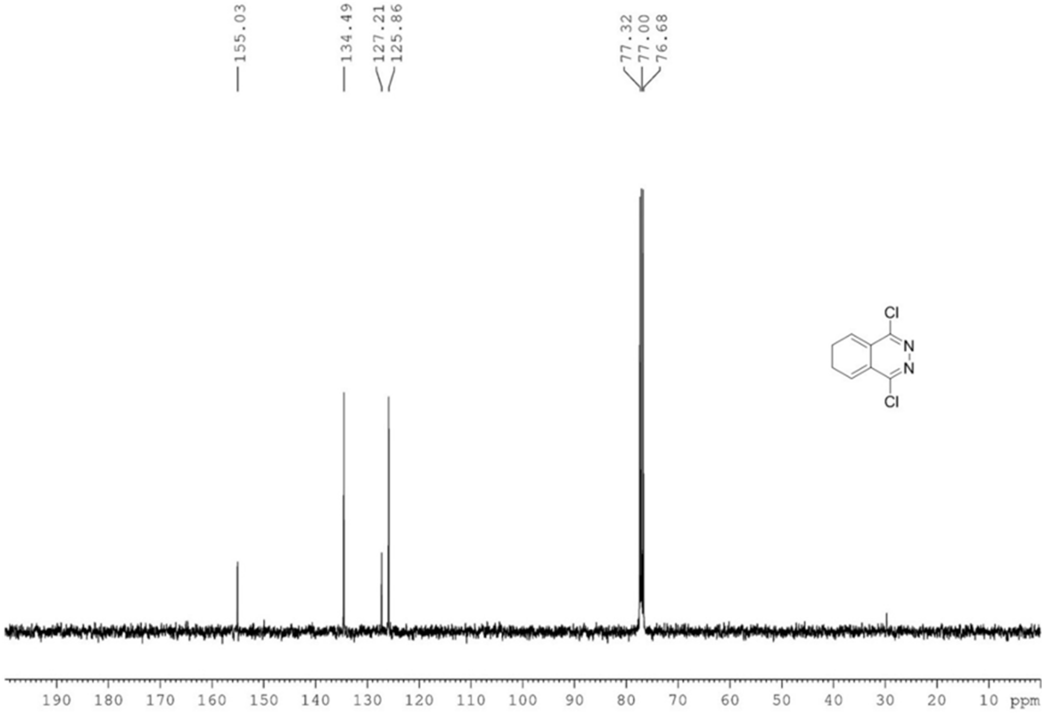

12

1,4-Dichloro-2,3-difluorophthalazine

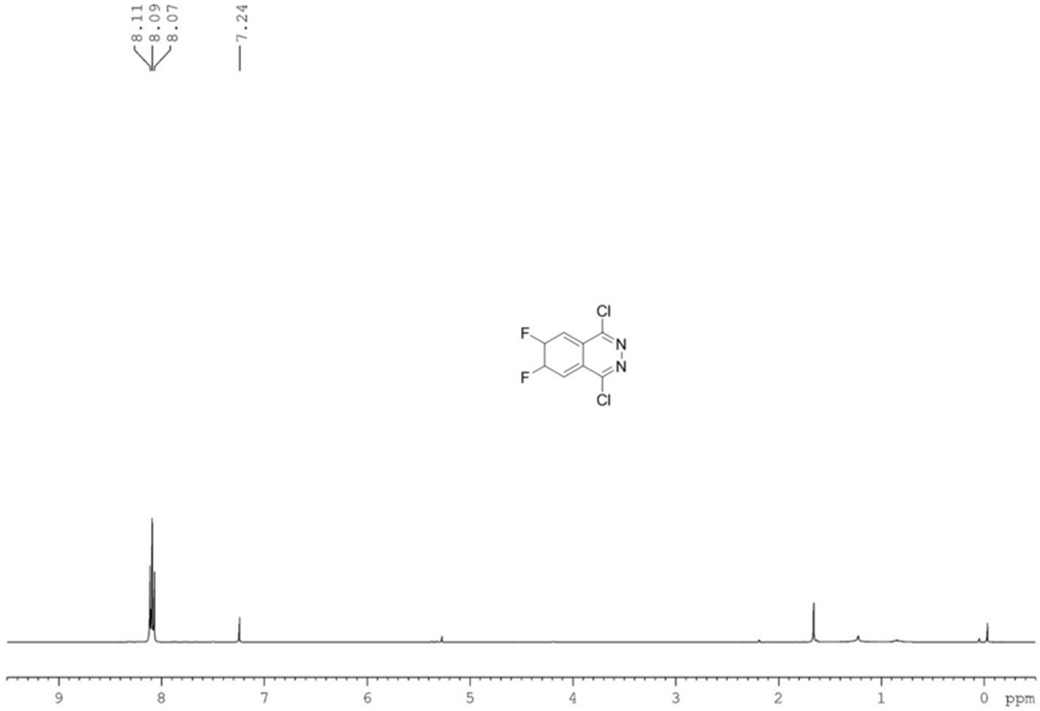

12

1,4-Dichloro-2,3-difluorophthalazine

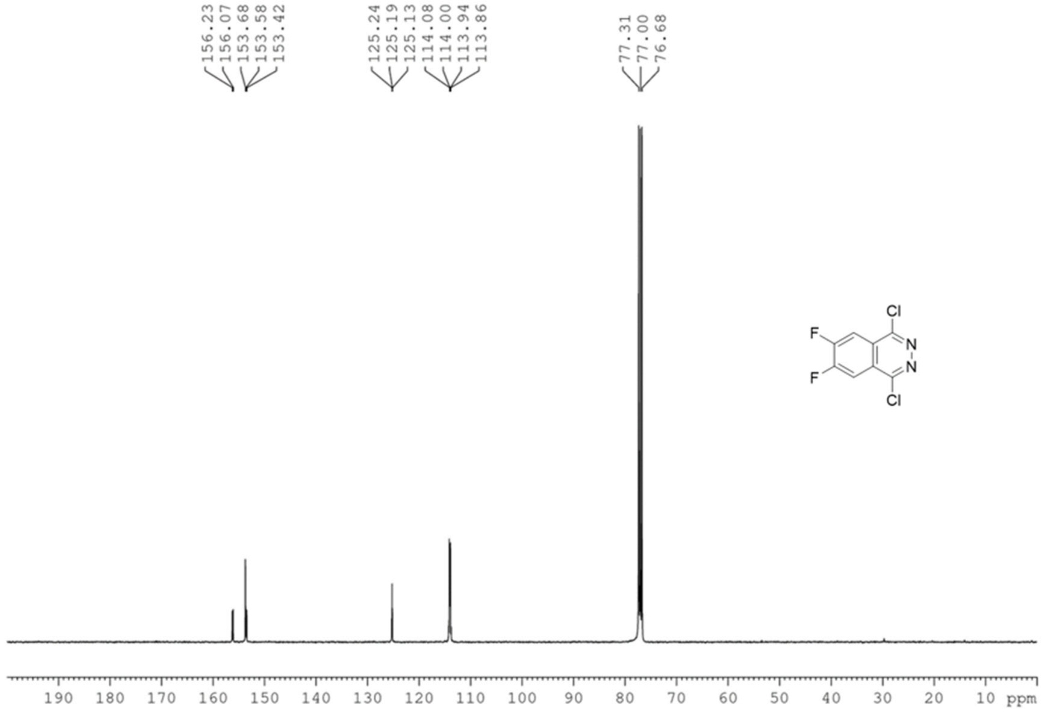

13

1,4,6,7-Tetrachlorophthalazine

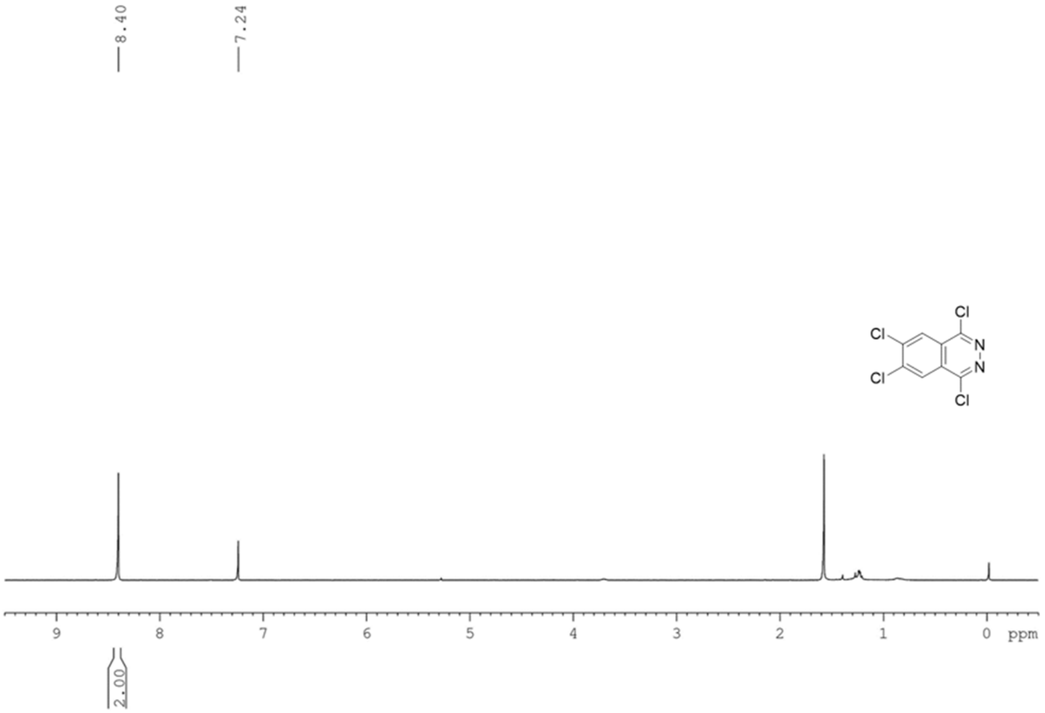

13

1,4,6,7-Tetrachlorophthalazine

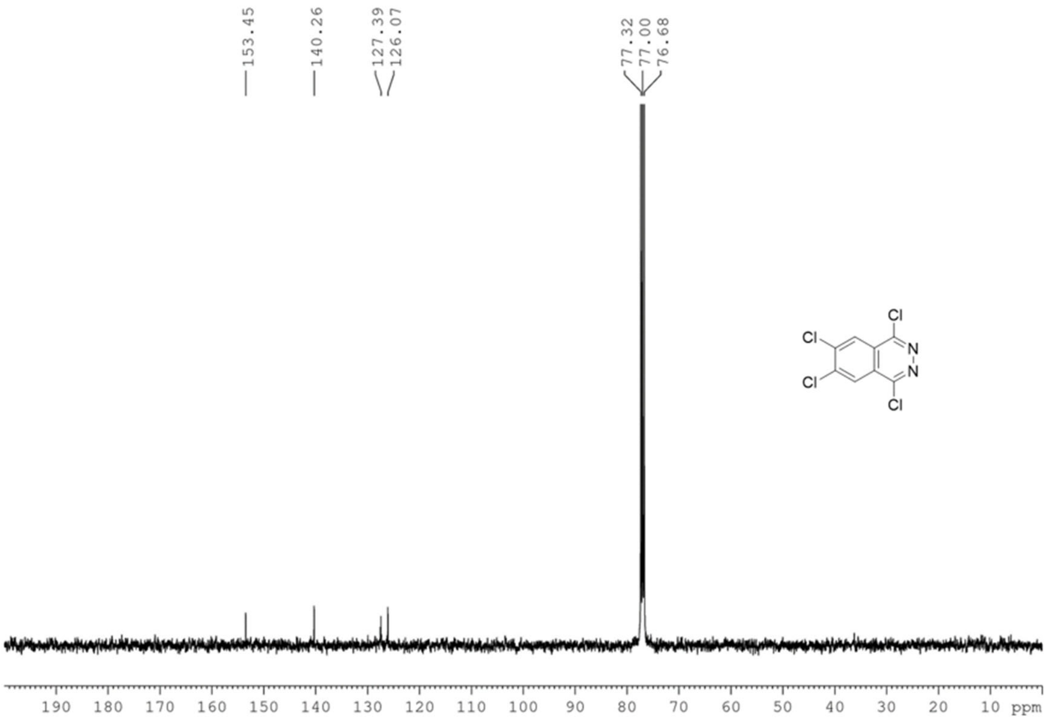

14

6,7-Dichloropyrazino[2,3-*d*]pyridazine

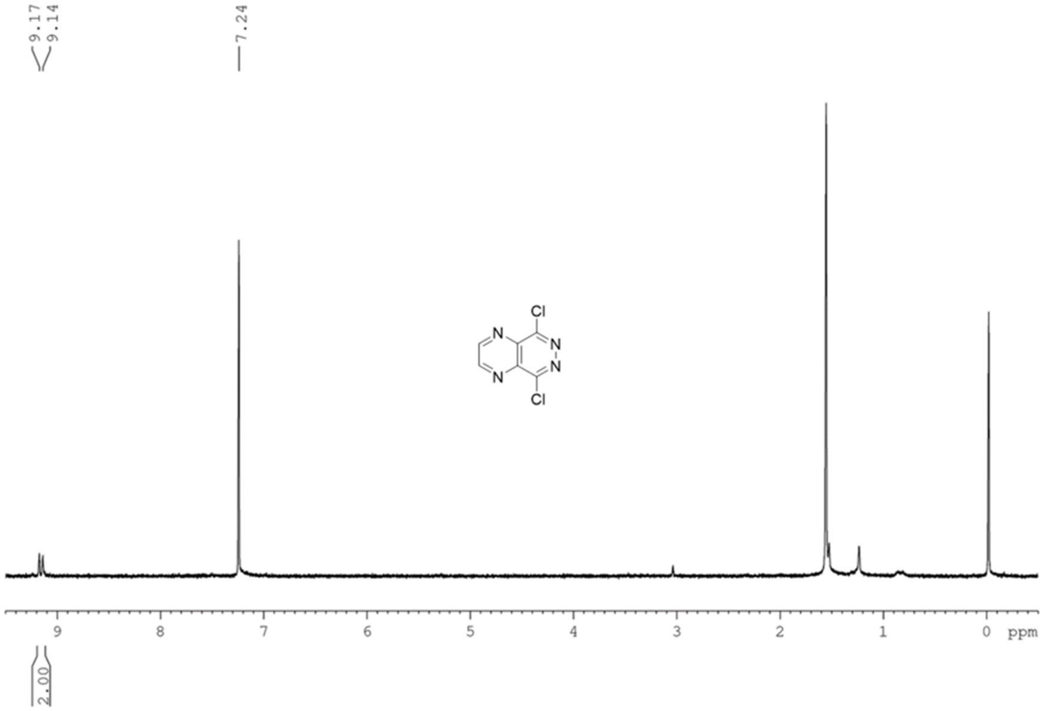

15

1,4-Dichlorobenzo[*g*]phthalazine

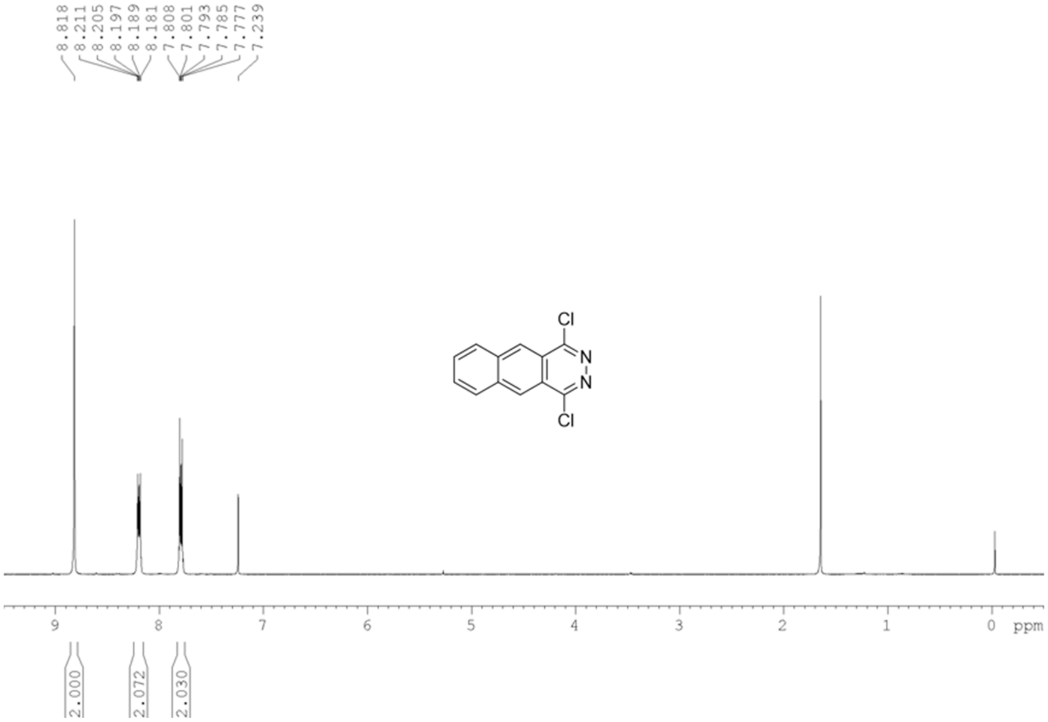

15

1,4-Dichlorobenzo[*g*]phthalazine

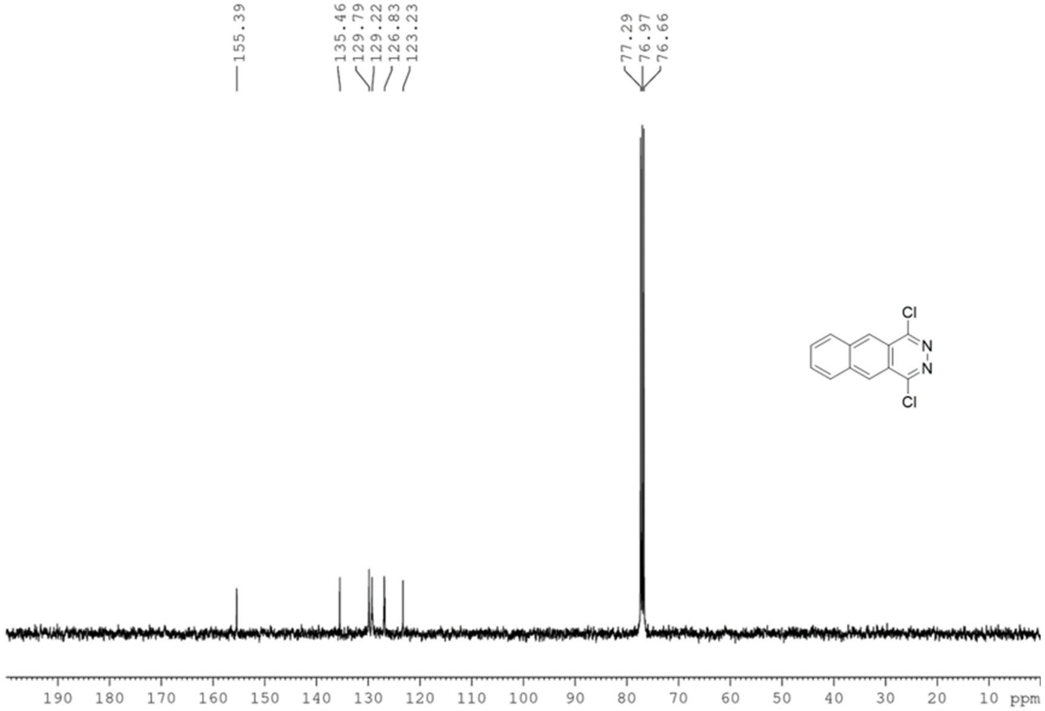

X-ray crystallography data section

ORTEP and Crystal structure determination of *N*'-(6,8-dioxo-1,3-diphenyl-6,8-dihydropyrazolo[3,4-*b*]pyrrolo[3,4-*d*]pyridin-7(3*H*)-yl)-*N*,*N*-dimethylformimidamide **4** (CCDC No. 1954819)

The crystal of **4** was obtained by slow evaporation of ethyl acetate and hexane (1:1). CCDC no: 1954819 (4) data may be obtained free of charge via www.ccdc.cam.ac.uk/data_request/cif, or by requesting Cambridge Crystallographic Data Centre, 12 Union Road, Cambridge CB2 1EZ, UK; fax: +44 1223 336033.

Table S1. Crystal data and structure refinement for *N'*-(6,8-dioxo6,8-dihydropyrazolopyrrolo-pyridine-yl)-*N*,*N*-dimethylformimidamide **4** (CCDC No. 1954819).

Empirical formula	C23 H18 N6 O2	
Formula weight	410.43	
Temperature	100(2) K	
Crystal system	Triclinic	
Space group	P -1	
Unit cell dimensions	a = 7.7714(5) Å	α= 91.624(3)°.
	b = 10.9660(6) Å	β= 94.362(3)°.
	c = 11.2302(6) Å	γ= 97.597(3)°.
Volume	945.19(9) $Å^3$	
Z	2	
Density (calculated)	1.442 Mg/m^3	
Absorption coefficient	0.097 mm^{-1}	

F(000)	428
Crystal size	0.20 x 0.20 x 0.1 mm^3
Theta range for data collection	1.820 to 26.475°.
Index ranges	-9<=h<=8, -13<=k<=13, -14<=l<=14
Reflections collected	12592
Independent reflections	3879 [R(int) = 0.0256]
Completeness to theta = 25.242°	99.6 %
Absorption correction	Semi-empirical from equivalents
Max. and min. transmission	0.7454 and 0.6737
Refinement method	Full-matrix least-squares on F^2
Data / restraints / parameters	3879 / 0 / 282
Goodness-of-fit on F^2	1.099
Final R indices [I>2sigma(I)]	R1 = 0.0371, wR2 = 0.0985
R indices (all data)	R1 = 0.0451, wR2 = 0.1128
Extinction coefficient	n/a
Largest diff. peak and hole	0.318 and -0.283 e.Å^{-3}
